# Maximizing ion accessibility in MXene-knotted carbon nanotube composite electrodes for high-rate electrochemical energy storage

**DOI:** 10.1038/s41467-020-19992-3

**Published:** 2020-12-02

**Authors:** Xiang Gao, Xuan Du, Tyler S. Mathis, Mengmeng Zhang, Xuehang Wang, Jianglan Shui, Yury Gogotsi, Ming Xu

**Affiliations:** 1 State Key Laboratory of Materials Processing and Die & Mold Technology, School of Materials Science and Engineering, Huazhong University of Science and Technology (HUST), 430074 Wuhan, P R China; 2 A. J. Drexel Nanomaterials Institute and Department of Materials Science and Engineering, Drexel University, Philadelphia, PA 19104 USA; 3 School of Materials Science and Engineering, Beihang University, 100083 Beijing, P R China

**Keywords:** Supercapacitors, Materials for energy and catalysis, Two-dimensional materials

## Abstract

Improving the accessibility of ions in the electrodes of electrochemical energy storage devices is vital for charge storage and rate performance. In particular, the kinetics of ion transport in organic electrolytes is slow, especially at low operating temperatures. Herein, we report a new type of MXene-carbon nanotube (CNT) composite electrode that maximizes ion accessibility resulting in exceptional rate performance at low temperatures. The improved ion transport at low temperatures is made possible by breaking the conventional horizontal alignment of the two-dimensional layers of the MXene Ti_3_C_2_ by using specially designed knotted CNTs. The large, knot-like structures in the knotted CNTs prevent the usual restacking of the Ti_3_C_2_ flakes and create fast ion transport pathways. The MXene-knotted CNT composite electrodes achieve high capacitance (up to 130 F g^−1^ (276 F cm^−3^)) in organic electrolytes with high capacitance retention over a wide scan rate range of 10 mV s^−1^ to 10 V s^−1^. This study is also the first report utilizing MXene-based supercapacitors at low temperatures (down to −60 °C).

## Abstract (web summary)

Improving the accessibility of ions in the electrodes of electrochemical energy storage devices is vital for charge storage and rate performance. Here, the authors report a new type of MXene-carbon nanotube composite electrode that maximizes ion accessibility, resulting in high rate performance at low temperatures.

## Introduction

The storage of charge in capacitive electrochemical energy storage devices begins with the adsorption of electrolyte ions on the surface or active sites of an electrode. Only the area of the electrode that is accessible to ions can contribute to the storage of charges. Therefore, ion accessibility can influence the capacitance or capacity of the energy storage devices^1,2^. The influence is more obvious when the devices are operated at high rates or low temperatures, especially in the case of organic electrolytes.

Since organic electrolytes can provide a wider working voltage window than aqueous electrolytes, organic-electrolyte-based energy storage devices are more promising for achieving high energy density^3^. The use of organic electrolytes is also essential for the operation of energy storage devices working at sub-zero temperatures. However, organic electrolytes have larger ions and lower conductivity than aqueous electrolytes. Besides, the solvation shells of ions in organic electrolytes also have a larger size than those in aqueous electrolyte. These properties hinder charge storage and slow the ion transport kinetics of electrode materials, limiting high-rate performances, especially at low temperatures^4–6^. Improving ion accessibility in organic-electrolyte-based energy storage devices, such as supercapacitors, remains a challenge^7^.

Efforts to improve ion accessibility for high-rate performance in organic electrolytes have been addressed using two approaches: electrolyte formulation and modification of electrode structure. As an example of electrolyte tuning, Wang et al.^8^ investigated how organic solvents influenced the intercalation of lithium ions into porous multilayer Ti_3_C_2_T_*x*_ and found that de-solvation had occurred in a lithium bis(trifluoromethylsulfonyl)imide/propylene carbonate (Li-TFSI/PC) electrolyte, which improved charge storage. A capacitance of 110 F g^−1^ (231 F cm^−3^) at 2 mV s^−1^ was reported and the scan rate range for their MXene-based supercapacitor was from 2 mV s^−1^ to 1 V s^−1^ with a capacitance retention of 58%. Yet only room-temperature properties were reported. Although the porous MXene used in this study facilitated the infiltration of the organic electrolytes in the electrodes at room temperature with the precise tuning of the electrolyte formulation, the low-temperature performance would still be limited because of the dramatically increased viscosity of electrolytes at low temperatures. Another study on tuning electrolyte composition showed that matching between the size of the electrode pores of carbon electrodes with the solvated ions of the electrolyte played an important role in achieving exceptional low-temperature performance^9^. A double-layer capacitance of 177 F g^−1^ at 5 mV s^−1^ was published with the rate performance from 5 to 500 mV s^−1^, while the capacitance retention of ~92.6% was obtained over the temperature range of 20 to −70 °C at 5 mV s^−1^. However, the heteroatoms (e.g., N, S, O) of the biomass-derived carbon limited the voltage window to 2.5 V, potentially lowering the energy density. Additionally, the rate performance data implied a limited ion transport and ion accessibility associated with the electrode structure.

When attempting to modify electrode structures themselves to improve ion accessibility, the common approaches include introducing pores in the surface of an electrode material through chemical etching^10,11^, increasing the interlayer spacing through modification of surface chemistry, or through the intercalation of pillaring materials to prevent restacking of two-dimensional (2D) sheets^12–15^. For example, Dall’Agnese et al.^16^ used multiwall carbon nanotubes (MWCNTs) as the spacers to separate MXene sheets and reported the capacitances ranging from 85 F g^−1^ (245 F cm^−3^) at 2 mV s^−1^ to 63 F g^−1^ (181 F cm^−3^) at 100 mV s^−1^. Another study reported MXene/MWCNT composite electrodes prepared by self-assembly of negatively charged, metallic Ti_3_C_2_T_*x*_ MXene flakes and positively charged CNTs as spacers. The capacitance of 75 F g^−1^ (175 F cm^−3^) at 10 mV s^−1^ was achieved, with the rate performance from 0.5 to 100 mV s^−1^ (ref. ^13^). Although the MXene/MWCNT composite electrodes produced by these approaches appear to have a larger interlayer spacing for their improved ion accessibility compared to unmodified MXenes, the scanning rate has never been achieved higher than 100 mV s^−1^ and only room-temperature properties have been reported. Even though these methods increase the spacing between the layers of the 2D electrode materials (e.g. MXene), the stacking problem persists that the 2D layers remain horizontally stacked. Therefore, the ion transport in the electrode is very tortuous and slow in the direction normal to the electrode surface, which would be an obstacle for high-rate performance and low-temperature operation. Recently, Sun et al.^17^ fabricated a graphene/Nb_2_O_5_ composite electrode by using a three-dimensional (3D) graphene framework as a conductive scaffold that resulted in high rate energy storage with a capacity retention of ~40% over a current density range of 0.2 to 20 A g^−1^. Although the Nb_2_O_5_ was not modified, i.e. the ion intercalation tunnels remained narrow and the electrical conductivity was low, the 3D graphene framework provided fast ion transport, leading to improved high-rate performance. As these studies show, in order to further improve the performance of energy storage systems at high rates, consideration must be given to both ion accessibility and ion transport.

In this study, we developed an approach to achieve high-rate performance in an organic electrolyte through the construction of a 3D electrolyte-accessible electrode structure. Ti_3_C_2_T_*x*_ (for simplicity we will write the chemical formula as Ti_3_C_2_ throughout the remainder of this paper) was chosen to form an interconnected network where the specially synthesized knotted CNTs were encased as the support for the Ti_3_C_2_ network. To simultaneously maximize ion accessibility and minimize the tortuosity of ion transport pathways, the structure of the electrodes was modified from being heavily stacked to being highly misaligned by optimizing the sizes and the mass ratios of the knotted CNTs and Ti_3_C_2_ flakes. The MXene-knotted CNT composite electrodes exhibited a high capacitance of 130 F g^−1^ (276 F cm^−3^) with a capacitance retention of ~56% over three orders of magnitude of scan rates from 10 mV s^−1^ to 10 V s^−1^. They also demonstrated superior stability in organic electrolytes without any capacitance loss upon 10,000 cycles. Our new electrodes even enabled impressive low-temperature operation. A high capacitance retention was obtained over the temperature range of 20 to −60 °C at 20 mV s^−1^. At −30 °C, the asymmetric device was capable of operating with a 4.2 V voltage window without any parasitic reactions or losses in capacitive behavior. It is the largest operating voltage window that has been reported so far for a MXene-based device. An energy density of 59 Wh kg^−1^ and a power density of 9.6 kW kg^−1^ were obtained at −30 °C, surpassing the best values reported for supercapacitors with 2D electrode materials operating at low temperatures.

## Results

### Design of MXene-knotted CNT composite electrodes

As discussed in the previous section, the mixing of nanomaterials (e.g. single- or multiwall CNTs, metal oxides) with MXenes has already been used to modify the interlayer spacing of MXene; however, the restacking of the MXene flakes still leads to a planar, paper-like electrode architecture with an in-plane orientation of the 2D sheets, severely limiting the accessibility of the electrolyte (Supplementary Fig. 1), which hinders the pseudocapacitive performance of MXenes at high scan rates in aqueous electrolyte and results in poor rate performance in organic electrolytes^18–21^.

To create the electrodes with a 3D electrolyte-accessible architecture, a special type of CNTs with large, knot-like structures with the size tens to hundreds of times larger than the typical nanomaterials that were used as spacer molecules were synthesized in order to misalign the Ti_3_C_2_ flakes in the electrode structure. We will refer to this special type of CNTs by calling them knotted CNTs. The knotted CNTs were produced by growing thick and thin CNTs in one assembly where the flexible thin CNTs were intertwined with stiff thick CNTs to form large knots. It should be noted that these large knots were not able to be produced by using CNTs with identical dimeters^13,22^. The thick CNTs were too stiff to form knots, and while the flexible thin CNTs can form knot-like structures, the size of the knots were not large enough to break the stacking of Ti_3_C_2_ flakes.

**Fig. 1:**
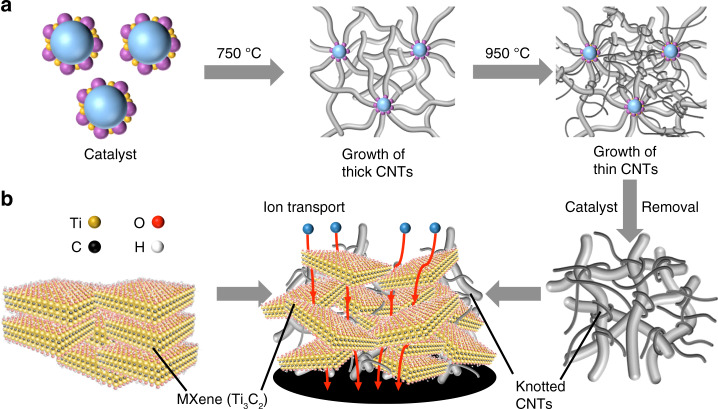
Design of the MXene-knotted CNT composite electrodes for efficient ion transportation. **a** Schematic of the synthesis process for the knotted CNTs, where thin CNTs were intertwined with thicker CNTs. **b** Schematic of a MXene-knotted CNT composite electrode with reduced horizontal orientation and lower ion transport tortuosity through the bulk of the electrode.

In order to simultaneously grow CNTs of different sizes, the catalyst nanoparticles with the catalysts of two different sizes were specially designed to facilitate the growth of the knotted CNTs (Fig. 1a). The catalyst nanoparticles were placed in a temperature-shifting two-stage fluidized bed reactor with separate temperature zones where the smaller catalyst produced thinner CNTs and the larger catalyst facilitated the growth of thick CNTs in the specific temperature zones (Supplementary Figs. 2 and 3 and Methods). Compared with the water-assisted chemical vapor deposition (CVD) method^23–26^, the knotted CNTs synthesized using the fluidized bed reactor had significant amounts of residual catalyst which needed to be removed via a purification possess. The MXene-knotted CNT composite electrodes were then prepared through a self-assembly process where Ti_3_C_2_ suspensions were mixed with knotted CNT solution before being filtered using vacuum assisted filtration to form freestanding films. A range of films with CNT contents (i.e. the mass ratio of CNTs to the electrode) from 5 to 34% were made and studied (Supplementary Table 1 and Supplementary Fig. 4). The thicknesses of the electrodes were controlled to be approximately 10 μm and all the electrodes displayed a mass loading higher than 1.8 mg cm^−2^. Detailed descriptions of the synthesis of the knotted CNTs and the electrode fabrication process are available in the *'*Methods' section.

Because of the mechanical constraints of the knotted CNTs, the large knotted structures were capable of maintaining the structural integrity of the electrode film while preventing the restacking of the MXene flakes during the electrode fabrication process, which has not been demonstrated previously (Fig. 1b). The structure of these composite electrodes ensures efficient ion transportation by dramatically reducing the tortuosity of the ion transport pathways.

**Fig. 2:**
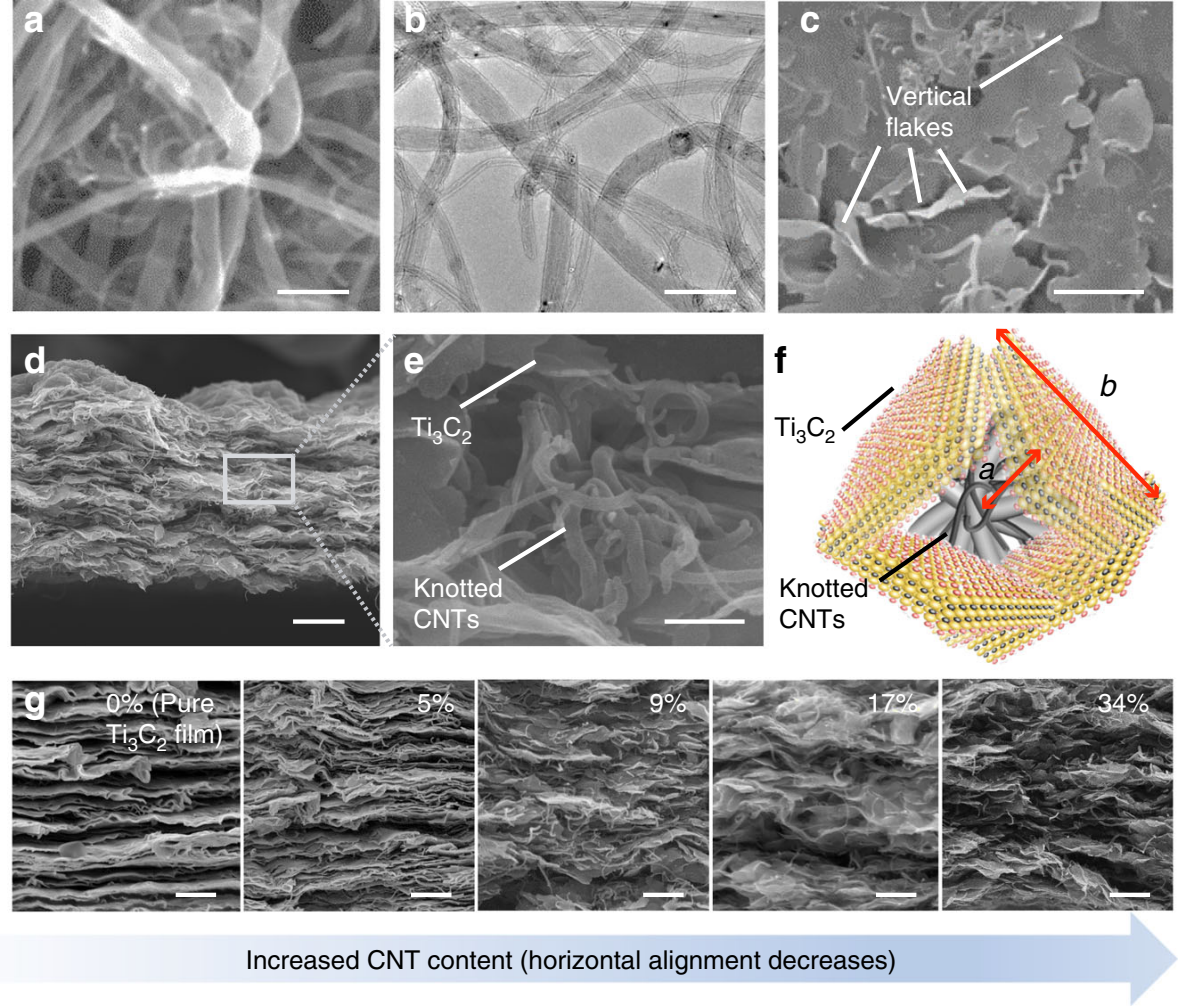
Characterization of the knotted CNTs and MXene-knotted CNT composite electrodes. **a** SEM and **b** TEM images of the knotted CNTs. **c** Top view and **d** cross-sectional SEM images of an MXene-knotted CNT composite electrode with 17% CNTs. **e** SEM image and **f** corresponding schematic of how the alignment of the Ti_3_C_2_ flakes was broken by the knotted CNTs to form 3D networked structure. “*a*” and “*b*” (~1:2 ratio) in the schematic correspond to the radius of the knots (taking the knots as circles) and the size of the Ti_3_C_2_ flakes, respectively. **g** Cross-sectional SEM images of MXene-knotted CNT composite electrodes with different CNT contents from 0% (pure Ti_3_C_2_ film) to 34%. Scale bars, 100 nm (**a**, **b**), 1 μm (**c**, **g**), 2 μm (**d**), 200 nm (**e**).

The successful synthesis of the knotted CNTs and the fabrication of the MXene-knotted CNT composite electrodes were confirmed by scanning electron microscopy (SEM) and transmission electron microscopy (TEM). Figure 2a showed the structure of the knotted CNTs, where the thin flexible CNTs (Ø = 15 ± 8 nm; length = 0.2–1 μm) were wound around the thick CNTs (Ø = 55 ± 5 nm; length = 5–15 μm), effectively forming large knots with sizes upwards of 200 ± 20 nm. To investigate their mechanical stability the knotted CNTs were sonicated at 500 W for 50 min. TEM images of the sonicated CNTs revealed that the cohesive structure of the CNT knots remained after sonication without a significant reduction in the size of the knots (Fig. 2b).

The breaking of the alignment of the MXene flakes by the knotted CNTs can be seen by looking at SEM images of the tops and cross-sections of the different MXene-knotted CNT composite electrodes (Fig. 2c, d). Figure 2c showed the porous morphology of the surface of the MXene-knotted CNT composite electrodes where several vertically oriented Ti_3_C_2_ flakes can be seen. The cross-sectional images showed how the MXene-knotted CNT composite electrodes had a significantly lower degree of horizontal orientation relative to the pure MXene film (Fig. 2d). In addition, the X-ray diffraction (XRD) patterns of the MXene-knotted CNT composite electrodes (Supplementary Fig. 5) showed that the orientation of the Ti_3_C_2_ flakes in the MXene-knotted CNT composite electrodes was more random since only the (002) peak was observable. None of the higher order (00ℓ) peaks that were characteristic of freestanding MXene films with highly aligned flakes were present. The (002) peaks were located at 8.19°, 6.03°, 5.61°, 5.22°, and 4.74° for the samples with CNT contents from 0% to 34%, respectively. The corresponding interlayer spacings calculated using the Bragg equation were 10.8, 14.7, 15.8, 16.9, and 18.6 Å^27,28^. Higher magnification SEM images (Fig. 2e) showed that the CNT knots were confined within the spaces between Ti_3_C_2_ flakes. The entirety of the electrode structure was composed of CNT knots encased by Ti_3_C_2_ flakes, exhibiting a 3D architecture with both increased interlayer spacing and misaligned MXene flakes. Compared to 2D stacked electrode structures that only had enlarged interlayer spacing, our 3D electrode structure provided increased open space for enhanced accessibility and reduced the tortuosity of the ion transport pathways to improve the transport of charge storing ions through the electrode.

It is important to note that correctly matching the sizes of the CNT knots and the Ti_3_C_2_ flakes is highly important for the successful formation of the 3D electrolyte-accessible architecture. Figure 2f showed a schematic for a small region of the MXene-knotted CNT composite electrode structure that illustrated how the size of the Ti_3_C_2_ flakes must be slightly larger than the CNT knots, ensuring that the misaligned Ti_3_C_2_ flakes form a continuous network with ample amounts of interstitial spaces. To achieve this structure (Fig. 2e, f), CNT knots were synthesized to be 200 ± 20 nm in size, while the Ti_3_C_2_ flakes that were used had an average flake size of ~250 nm (Supplementary Fig. 6). Smaller CNT knots were not capable of breaking the alignment of the Ti_3_C_2_ flakes while CNT knots that were too large size damaged the conductive network of the MXene flakes (Supplementary Fig. 7), resulting in the electrode having poor electrical conductivity.

The CNT content also plays a role in the formation of the 3D electrolyte-accessible architecture. As shown in Fig. 2g, increasing the knotted CNT content resulted in the architecture of the electrodes transitioning from a stacked, planar structure to a 3D networked architecture. When the CNT content was small (5%), negligible variations in the electrode structure occurred, and the cross-sectional image of the 5% CNT electrode showed that the film was still highly aligned. Significant variations were only observed when the CNT content was increased to 9%. However, the 9% CNT electrode still had a relatively densely packed structure with some regions of stacked MXene flakes visible. The structure of the 17% CNT electrode was noticeably less packed, while the cross-sectional image of the 34% CNT electrode showed there were still some regions of densely stacked Ti_3_C_2_. Incorporating higher amounts of knotted CNTs likely led to the separation of the MXene and CNT phases within the electrode structure due to the significant difference in the densities of the CNTs and Ti_3_C_2_, which would affect the conductivity of the electrodes. The sheet resistance of the different MXene-knotted CNT composite films decreased from 0.15 to 10 Ω per square as the CNT content increased from 0% to 34% (Supplementary Table 1).

### High-rate electrochemical performance in an organic electrolyte

**Fig. 3:**
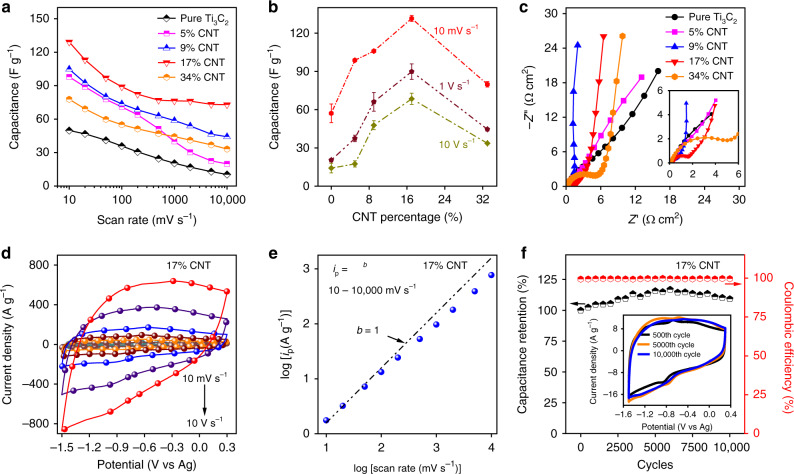
Electrochemical analysis of the MXene-knotted CNT composite electrodes in an organic mixed electrolyte (EMIM-TFSI: Li-TFSI = 1:1). **a** Rate performance of the electrodes with different CNT contents from 0% (pure Ti_3_C_2_). **b** Capacitance of the electrodes as a function of CNT content at 10 mV s^−1^, 1 V s^−1^, and 10 V s^−1^. The error bars represent the standard deviation. **c** Electrochemical impedance spectroscopy (EIS) data for the different electrodes taken at 0 V versus open circuit voltage. The inset showed a magnified view of the high-frequency region. **d** Cyclic voltammograms at different scan rates, **e** plot of the anodic peak current versus the scan rate, and **f** long-term cycling performance and corresponding Coulombic efficiency at 100 mV s^−1^ for the 17% CNT electrode.

To investigate the electrochemical performance of the MXene-knotted CNT composite electrodes, we first evaluated the electrodes in 1 M 1-ethyl-3-methylimidazolium bis(trifluoromethylsulfonyl)imide/ acetonitrile (EMIM-TFSI/ACN). Although the electrolyte contained a large cation (EMIM), the MXene-knotted CNT composite electrodes showed a capacitance of 63 F g^−1^ at 10 mV s^−1^ and good rate performance with a capacitance retention of 56% from 10 mV s^−1^ to 2 V s^−1^ (Supplementary Fig. 8a, b). To further improve the capacitance, lithium bis(trifluoromethylsulfonyl)imide (Li-TFSI) was mixed with the EMIM-TFSI/ACN (EMIM-TFSI: Li-TFSI = 1:1) as the Li ions can intercalate into the Ti_3_C_2_ resulting in increased charge storage. Furthermore, the dissimilar cations in the electrolyte were also beneficial for decreasing the freezing point of the electrolyte to improve low-temperature operation^29,30^. The cyclic voltammograms (CVs) at different potential windows were tested at 10 mV s^−1^ and displayed a sharp irreversible peak at the potential lower than −1.5 V versus Ag (Supplementary Fig. 8c). Therefore, the working potential window in the mixed electrolyte was finally chosen to be −1.5 to 0.3 V. Figure 3a, b showed the rate performance of the MXene-knotted CNT composite electrodes with different CNT contents ranging from 0% (pure Ti_3_C_2_ film) to 34% in the mixed electrolyte (EMIM-TFSI/Li-TFSI/ACN). While the pure Ti_3_C_2_ electrode (0% CNTs) had a specific capacitance of 50 F g^−1^ at 10 mV s^−1^ and a capacitance retention of only 20% from 10 mV s^−1^ to 10 V s^−1^, the MXene-knotted CNT composite electrode with 17% CNTs had significantly improved specific capacitance and rate performance with ~130 F g^−1^ at 10 mV s^−1^ and ~73 F g^−1^ at 10 V s^−1^, approximately 56% of the initial capacitance value at 10 mV s^−1^, surpassing most of the best values that have been reported for the MXene-based electrodes tested in organic electrolytes^12,15,16^. In all, 130 F g^−1^ (~276 F cm^−3^) is also the highest reported capacitance for Ti_3_C_2_ MXene-based electrodes for electrolytes using acetonitrile as the solvent (Supplementary Fig. 9)^8,16^.

The increased rate performance with the increased CNT content up to 17% was attributed to a transition of the structure of the electrode from a stacked, planar structure to a 3D networked architecture, as described previously. The decreases in capacitance and capacitance retention occurred when the CNT content was increased to 34%, which was likely due to the larger internal resistance of the 34% CNT electrode caused by the discontinuous MXene network (Fig. 2g and Supplementary Table 1). Moreover, we prepared a composite electrode by mixing MXene with commercially available non-knotted MWCNTs for comparison (Supplementary Fig. 10). As shown by Supplementary Fig. 10a, b, the non-knotted MWCNTs possessed a uniform diameter of ~35 nm and the electrode displayed a 2D stacked structure, which was very different from the 3D electrolyte-accessible electrodes structure formed by our knotted CNTs. The MXene/non-knotted MWCNT electrode only showed a capacitance retention of 39% from 10 to 500 mV s^−1^ (Supplementary Fig. 10c, d). The results indicated that the ion transport in the MXene/non-knotted MWCNT electrode remained tortuous and the increases in ion accessibility were still limited even when the interlayer spacing of the electrode was increased compared to the pure MXene. It is clear that the construction of a 3D electrolyte-accessible electrode structure through the use of specially synthesized knotted CNTs has a significant effect on both reducing the tortuosity of ion transport and increasing ion accessibility.

Electrochemical impedance spectroscopy (EIS) measurements were performed using each of the MXene-knotted CNT composite electrodes and the pure MXene electrode at open circuit voltage (OCV). As shown in Fig. 3c, the resulting Nyquist plots for the MXene-knotted CNT composite electrodes with the CNT contents higher than 9% showed significant changes in the low-frequency region relative to the MXene pure film, with each electrode showing nearly vertical lines normal to the real axis (*Z*′), which was consistent with the improved ion accessibility and transport in the electrodes with higher knotted CNT loading^31,32^. In contrast, the Nyquist plots of the pure Ti_3_C_2_ MXene electrode and the 5% CNT electrode both showed a nearly constant increase in the imaginary and real impedance, indicating the sluggish ion diffusion for these densely packed electrodes. The evolution of the Nyquist plots revealed how breaking the stacking of the Ti_3_C_2_ flakes was necessary for improving the electrochemical kinetics.

A full electrochemical evaluation was conducted to further evaluate the full capabilities of the 17% CNT electrode as the best performing MXene-knotted CNT composite electrode. Figure 3d showed CVs for the 17% CNT electrode from 10 mV s^−1^ to 10 V s^−1^. In the plot of capacitance versus potential derived from cyclic CVs (Supplementary Fig. 8d), two de-intercalation peaks can be seen at −0.45 and −0.62 V versus Ag in the anodic scan. The two peaks may correspond to the de-intercalation processes for the EMIM and Li ions^8,16^. The corresponding cathodic peaks showed small peak separation, indicating that the charge storage process had a fast, non-diffusion-limited intercalation mechanism^33,34^. The coulombic efficiency increased from 94.4 to 99.4% at the scan rate increasing from 10 to 200 mV s^−1^ and maintained at ~100% at the high scan rate from 200 to 10,000 mV s^−1^ (10 V s^−1^) (Supplementary Fig. 8e). Additionally, the X-ray photoelectron spectroscopy (XPS) spectra of the MXene-knotted CNT electrodes before and after cycling showed that the surface chemistry was not noticeably altered, indicating that the electrochemical reactions did not significantly affect the surface chemistry of the MXene in this system (Supplementary Fig. 11). These results were consistent with the nonlinear curves of the galvanostatic charge–discharge (GCD) tests shown in Supplementary Fig. 12 and the impedance spectra recorded at different applied potentials (Supplementary Fig. 13). As shown in Fig. 3e, the analysis of the dependence of the peak current on the scan rate indicated that the *b* value of the 17% CNT electrode was close to 1 over a large scan rate range of 10 to 10,000 mV s^−1^, which was characteristic of non-diffusion limited pseudocapacitive charge storage^8^. Long-term cycling stability of the same electrode tested at 100 mV s^−1^ showed that the MXene-knotted CNT composite electrode was highly stable with almost no capacitance decay observable after 10,000 cycles (Fig. 3f) with a consistent coulombic efficiency near ~100%. As addressed above, the structural stability of the knotted CNTs can even withstand sonication due to the mechanical constraints (Fig. 2b). The knotted CNTs were capable of maintaining the structural integrity of the electrode film by preventing the restacking of the MXene flakes upon charge/discharge, which guaranteed the efficient ion transportation during long-term cycling.

### Low-temperature operation of full cell devices

**Fig. 4:**
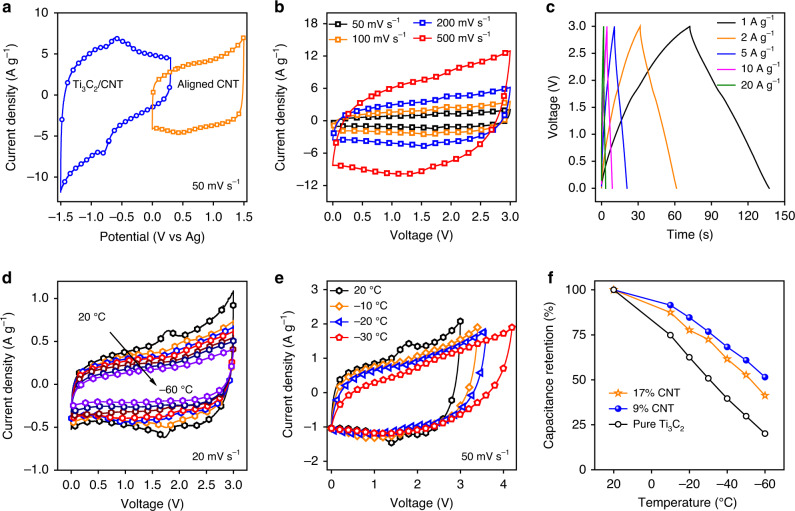
Electrochemical tests for asymmetric full cells. **a** Full cell fabrication: three-electrode CVs of the MXene-knotted CNT (Ti_3_C_2_/CNT) composite electrode and aligned CNT electrode at 50 mV s^−1^. **b** Cyclic voltammograms for the full cell at different scan rates. **c** Galvanostatic charge–discharge curves for the full cell at different current densities. **d** Cyclic voltammograms at different temperatures at 20 mV s^−1^ and **e** cyclic voltammograms with larger voltage windows at low temperatures for the full cell using MXene-knotted CNT composite electrode with a CNT content of 17% as the negative electrode. **f** Capacitance retention of full cells with different Ti_3_C_2_ MXene-based electrodes as a function of temperature.

Electrochemical energy storage at low temperatures is challenging due to the reduced mobility and transport of electrolytes near their freezing point. Using a highly conductive electrode material like Ti_3_C_2_ MXene with improved electrolyte accessibility can potentially mitigate the negative effects of lower temperatures. Since MXene would be oxidized at positive potentials and cannot be used as a positive electrode, we assembled asymmetric full cells with a MXene-knotted CNT composite electrode as the negative electrode and an aligned CNT electrode as the positive electrode to evaluate the performance of the MXene-knotted CNT composite electrodes at low temperatures (Fig. 4a–c and Supplementary Figs. 14 and 15). The aligned CNT electrode showed a working potential of 1.5 V as the positive electrode (Fig. 4a). The voltage window of the cell was maximized by balancing the mass ratio between the positive and negative electrodes. A voltage window of 3 V was realized with a mass ratio between the positive and negative electrodes of ~3:2. The capacitance of the aligned CNT electrode (94 F g^−1^ at 10 mV s^−1^) was found to be lower than that of the MXene-knotted CNT composite electrode (~130 F g^−1^ at 10 mV s^−1^) (Supplementary Fig. 14). Since the capacitance of the cell was limited by the lower-capacitance electrode, the aggregate capacitance can be further improved by using the positive electrode possessing higher capacitance. The CVs showed that a set of reversible redox peaks were still present in the full cell with negligible decreases in the capacitance occurring when the scan rate increased from 50 to 500 mV s^−1^ (Fig. 4b and Supplementary Fig. 15a**)**. The GCD curves in Fig. 4c were symmetric with no plateau from 1 to 20 A g^−1^, indicating the pseudocapacitive mechanism. Moreover, the cell also showed good stability with a capacitance retention of 90% after 8000 cycles (Supplementary Fig. 16).

Figure 4d showed the CV curves of the asymmetric cell with a 17% CNT electrode as the negative electrode at 20 mV s^−1^ from 20 to −60 °C. At −10 °C the asymmetric cell had a capacitance of ~20 F g^−1^, and approximately 50% of the cell capacitance (relative to 20 °C) was retained at −50 °C with the CVs still showing the capacitive behavior at the lower temperatures (Supplementary Fig. 15b). The shape of the CV curves became similar to what would be expected from a classic electrochemical double-layer capacitor as the redox/intercalation peaks disappeared. It is safe to assume that the double-layer component of the charge mechanism of these electrodes dominates at sub-zero temperatures.

Since electrochemical reactions are suppressed at low temperatures, a larger operating potential window should be possible at lower temperatures without the occurrence of any harmful side reactions (Fig. 4e). The highest cell voltage we achieved was 4.2 V with a coulombic efficiency of 97% at −30 °C, significantly larger than the voltage windows of previously reported asymmetric MXene-based supercapacitor systems. Moreover, the capacitance remained approximately the same when the voltage window was increased, leading to a significantly improved energy density at lower temperatures. Supplementary Fig. 17 showed the relationship between the energy and power densities normalized to the mass of both electrodes at low temperatures for the asymmetric cell. An impressive energy density of 59 Wh kg^−1^ and a power density of 9.6 kW kg^−1^ were obtained at −30 °C, surpassing the best values reported for supercapacitors with 2D electrode materials operating at low temperatures^35–40^.

We compared the performance of our MXene-knotted CNT composite electrode with the electrodes based on commercial high surface area activated carbon (AC). The AC-based symmetric cell showed a capacitance of 24 F g^−1^ at 10 mV s^−1^ and a capacitance retention of 29% from 10 to 500 mV s^−1^. In addition, the capacitance of the AC-based symmetric cell decreased from 23 to 1.5 F g^−1^ at 20 mV s^−1^ when the temperature decreased from 20 to −60 °C (Supplementary Fig. 18). As seen in Supplementary Fig. 17, our cell has a comparable capacitance of ~23 F g^−1^ with the AC-based cell, yet has much improved capacitance retention at increased scan rates (50% from 10 to 500 mV s^−1^) and at low temperatures (55% from 20 to −60 °C) (Fig. 4f). Moreover, our cell had a larger voltage window (4.2 V) than that of the AC-based symmetric cells (3 V) at low temperatures, which delivered energy density of 59 Wh kg^−1^ that was almost four times the value of the AC-based cell (~15 Wh kg^−1^).

The low-temperature performances of the asymmetric cells with the MXene-knotted CNT composite electrodes with different CNT contents are shown in Fig. 4f and Supplementary Fig. 19. Although the asymmetric cell with the 17% CNT electrode as the negative electrode had the highest capacitance of 20 F g^−1^ at low temperatures (Supplementary Fig. 19), the asymmetric cell with the 9% CNT electrode had the highest capacitance retention of ~55% over the temperature range of 20 to −60 °C. This result suggested that the electronic conductivity of the electrodes also played an important role during low-temperature operation. The Nyquist plots of the cell with the 17% CNT electrode exhibited almost identical curves from 20 to −60 °C, indicating the temperature-independent capacitive behavior (Supplementary Fig. 20a). Conversely, the Nyquist plots of the asymmetric cell with pure Ti_3_C_2_ MXene as the negative electrode were severely distorted to the right at low temperatures (Supplementary Fig. 20b). This deviation from the ideal capacitive behavior matched the rapid decrease of capacitance at lower temperatures for the asymmetric cell with the pure Ti_3_C_2_ electrode (Fig. 4f). In addition, when the temperature decreased from 20 to −60 °C, the equivalent series resistance (ESR) values of the cell with the 17% CNT electrode and the cell with the pure Ti_3_C_2_ film increased from 0.28 to 1.91 Ω cm^2^ and from 0.24 to 1.35 Ω cm^2^, respectively (Supplementary Fig. 20c, d). It can be inferred that the increase of ESR values was associated with the lowered ionic conductivity of the electrolyte at low temperatures (Supplementary Fig. 21).

Although the pure Ti_3_C_2_ electrode had higher conductivity, the MXene-knotted CNT composite electrodes performed better at lower temperatures. From these results, it is clear that the architecture and ionic conductivity of the electrode are key factors for developing capacitive energy storage systems capable of operating at low temperatures where electrolyte kinetics are limiting.

## Discussion

We have demonstrated how the architecture of an electrode can lead to enhanced rate performances in organic electrolytes. By using specially designed knotted CNTs, the MXene-knotted CNT composite electrodes showed a high capacitance retention of ~56% over a scan rate range of 10 mV s^−1^ to 10 V s^−1^ in a mixed organic electrolyte. The large knot-like structures in the knotted CNTs were essential for disrupting the alignment of the Ti_3_C_2_ flakes, leading to the ion transport pathways with lower tortuosity and enhanced ion accessibility. The advantages of the electrode resulted in improved low-temperature operation for a Ti_3_C_2_ MXene-based supercapacitor, with a high capacitance retention of ~55% when the operating temperature was decreased from 20 to −60 °C. This study has demonstrated that pseudocapacitive materials can be used for energy storage at high rates in organic electrolyte after tailoring the design of the electrode architecture.

## Methods

### Preparation of Ti_3_C_2_ solution

Multilayer Ti_3_C_2_ was produced by etching Ti_3_AlC_2_ MAX phase (Carbon-Ukraine) in an acidic fluoride containing etchant^41^. One gram of Ti_3_AlC_2_ was slowly added to 20 mL of the etchant solution which contained 12 mL of 9 M HCl, 2 mL of 49% HF, and 6 mL of H_2_O. The mixture was stirred at 35 °C for 24 h using a hot plate. The multilayer Ti_3_C_2_ was washed with deionized water by repeated centrifugation and decantation until the pH of the supernatant was neutral. The sediment was dispersed in 50 mL of deionized water and delaminated by adding 1 g LiCl. The solution was then stirred for 6 h and then washed for four times with deionized water by centrifuging at 3500 r.p.m. The obtained single layer Ti_3_C_2_ was then dispersed in deionized water and sonicated for 1 h with Ar bubbling in an ice bath to reduce the size of the flakes. After centrifuging of the sonicated solution at 3500 r.p.m. for 1 h, the final Ti_3_C_2_ solution in water was collected as the dark supernatant.

### Preparation of knotted CNTs

Ni–Mn–Al–O catalysts were synthesized using a typical co-precipitation and subsequent calcination reaction. First, Ni(NO_3_)_3_, Mn(NO_3_)_2_, and Al(NO_3_)_3_ were dissolved in 200.0 mL deionized water with [Ni^2+^] + [Mn^2+^] + [Al^3+^] = 0.2 mol L^−1^, n(Ni):n(Mn):n(Al) = 0.4:1:2. Then, 50 mL 2.5 mol L^−1^ NaOH was added into the solution and the solution was then left to stand at 95 °C for 12 h in a 500 mL flask, which was equipped with a reflux condenser in ambient atmosphere. The as-obtained suspension was filtered, washed by deionized water, and freeze-dried to get the NiMnAl LDHs. The NiMnAl LDHs were then loaded into a quartz boat and calcinated at 700 °C for 2 h in a quartz tube to get the final product Ni–Mn–Al–O.

The knotted CNTs were prepared using a temperature shift two-stage fluidized bed. In detail, 0.2 g Ni–Mn–Al–O nanoparticles were loaded into the reactor and processed with our customed two-step growth (Supplementary Fig. 2). The first-step growth was held in the upper zone of the reactor with the temperature of 750 °C. The nanoparticles were immersed in the H_2_ (flow rate of 80 mL min^−1^) for 10 min for the catalyst formation and then C_2_H_2_ (flow rate of 100 mL min^−1^) was introduced for growing CNTs for 15 min. During the first-step growth, the large-sized catalysts were conceived from the nanoparticles and thick CNTs were acquired with the average diameter of ~55 nm (Supplementary Fig. 3a). Then the isolation board was opened and the nanoparticles (with the thick CNTs on them) were moved to the lower zone of the reactor with the temperature of 950 °C to proceed the second-step growth for another 15 min. During this process, the small catalysts were conceived from the nanoparticles and the thin CNTs were grown with the average diameter of ~15 nm (Supplementary Fig. 3b). Then the knotted structured CNTs with a bimodal diameter distribution were obtained. The furnace was cooled to room temperature under Ar protection. The as-grown products were collected and purified by routine HCl (5 mol L^−1^) and NaOH (13 mol L^−1^) treatment.

### Preparation of electrodes

For the preparation of MXene-knotted CNT composite electrodes, the knotted CNTs were first dispersed in a CTAB solution (0.1 wt%) by probe sonication to get a CNT concentration of 0.5 mg mL^−1^. MXene-knotted CNT composite electrodes were prepared by a self-assembly process. Typically, a CTAB-grafted CNT solution was added dropwise to a Ti_3_C_2_ MXene suspension with varying CNT contents from 5% to 34%. The mixture was probe sonicated for 10 min and then filtered using a Celgard membrane (3501). MXene-knotted CNT composite electrodes were obtained after washing with deionized water and vacuum drying at 80 °C.

For comparison, MXene-MWCNT composite electrodes were prepared by using the same self-assembly method except for using the commercially available non-knotted MWCNTs (Shenzhen NANO Tech. Port. Co. Ltd, China). The AC electrodes were prepared by mixing 80 wt% AC (YP50, Kuraray chemical, Japan), 10 wt% acetylene black (Alfa Aesar, 99.9%), and 10 wt% poly(tetrafluoroethylene) (PTFE, Aladdin, 60%) as a binder in ethanol. The solution was stirring overnight, and ethanol was then evaporated at 80 °C, forming a rubber-like paste. The paste was rolled into a film for use. The mass loading of the AC electrode was ~2 mg cm^−2^.

The aligned CNT electrode was prepared by directly using the aligned CNT forest that was synthesized by the water-assisted CVD method as the positive electrodes. For the synthesis of the aligned CNT forest, Al_2_O_3_ (~30 nm)/Fe (~2 nm) metal layers were firstly sputtered on an Si substrate with an oxide layer (600 nm). Then, the catalyst coated substrate was inserted into a quartz tube furnace under flowing Ar and H_2_ (total flow 1000 mL min^−1^). When reaching the reaction temperature of 750 °C, C_2_H_4_ was introduced into the furnace with a flow rate of 100 mL min^−1^. The CVD growth was maintained for 15 min at 750 °C with a water vapor concentration of ~75 ppm.

### Material characterization

The morphology of Ti_3_C_2_ MXene and MXene-knotted CNT composite electrodes were observed using SEM (Zeiss Surra 50VP, Germany or FE-SEM, FEI Nova 450 Nano) and TEM (JEOL JEM-2100, Japan). Structure of the Ti_3_C_2_ MXene and MXene-knotted CNT composite electrodes were characterized by XRD (SmartLab, Rigaku 112 Corp. or X’Pert Pro, PANanalytical) using Cu Kα_1_ radiation. Surface chemistry of the MXene-knotted CNT composite electrodes was characterized by XPS (ESCALab 250).

### Electrochemical tests

Electrochemical measurements were performed in three-electrode Swagelok-type cells. Pure Ti_3_C_2_ MXene electrodes prepared by vacuum filtration and MXene-knotted CNT composite electrodes were used directly as the working electrodes and AC films (YP50, Kuraray) with 5% PTFE were used as the counter electrodes. A silver wire was used as the reference electrode. Polypropylene membranes (Celgard 3501) were used as the separators, and 1 M solution of EMIM-TFSI/Li-TFSI (EMIM-TFSI: Li-TFSI = 1:1) in ACN or 1 M EMIM-TFSI/ACN was used as the electrolyte. For the asymmetric cells, aligned CNTs were chosen as the positive electrode and assembled with the MXene-knotted CNT composite electrodes in the Swagelok-type cells. Electrochemical measurements of GCD, cyclic voltammetry and EIS were performed using a VMP3 potentiostat (BioLogic). Scanning rates ranging from 10 mV s^−1 ^to 10 V s^−1^ were used for the cyclic voltammetry experiments with a working potential window of 1.8 V (−1.5 to 0.3 V versus Ag). The impedance spectroscopy analysis was performed from 10 mHz to 200 kHz from OCV to the maximum applied voltage. Cycling stability was tested at a scan rate of 100 mV s^−1^ for 10,000 cycles. The low-temperature tests were conducted in a high/low-temperature test chamber (MCB-1.2-.33-.33-H/AC). The ionic conductivity of the electrolyte at different temperature was tested by using the conductivity isopod (EDAQ/EPU357).

### Calculations for the electrochemical tests

The capacitance of a single electrode (*C*_sp_) in the three-electrode cell was calculated from the anodic scan of CV curve based on

1 $$C_{{\mathrm{sp}}} = \frac{{\mathop {\smallint } {\it{i}} \cdot {\mathrm{d}}t}}{{{\it{m}} \cdot {\it{V}}}},$$

where *i* was the discharging current, *m* was the mass of the working electrode and *V* was the voltage window (1.8 V) of the CV scan.  *C*_sp_ was also calculated from the discharging curve of GCD for comparison.

For the two-electrode cell, the capacitance of the full cell (*C*_cell_) was calculated from the cathodic scan of the CV curves:

2 $$C_{{\mathrm{cell}}} = \frac{{\mathop {\smallint } {\it{I}} \cdot {\mathrm{d}}{\it{t}}}}{{m_{{\mathrm{total}}} \cdot V_{{\mathrm{cell}}}}},$$

where *m*_total_ was the whole mass of both electrodes and *V*_cell_ was the voltage window of the full cell.

The energy density of the full cell was estimated based on the discharging curves of the GCD:

3 $$E_{{\mathrm{cell}}} = I\left( {{\int}_0^{t_{\mathrm{d}}} {{\it{V}}{\mathrm{d}}t} } \right),$$

where *I* was the discharging current density, *t*_d_ was the discharging time and *V* was the voltage of the asymmetric cell. The power density was further evaluated based on *P* = *E*_cell_/Δ*t*.

## Supplementary information

Supplementary Information

Peer Review File

## Data Availability

The data that support the plots within this paper and other finding of this study are available from the corresponding author upon reasonable request.
