# Supplementary material for: Maximizing ion accessibility in MXene-knotted carbon nanotube composite electrodes for high-rate electrochemical energy storage
Source: Nat Commun. 2020 Dec 2;11:6160. doi: 10.1038/s41467-020-19992-3 (PMC7710708; doi:10.1038/s41467-020-19992-3)
Supplement: Supplementary file 1 — Supplementary Information [file 41467_2020_19992_MOESM1_ESM.pdf]

Maximizing ion accessibility in MXene-knotted carbon nanotube composite electrodes for high-rate electrochemical energy storage

Xiang Gao,[a#] Xuan Du,[a#] Tyler S. Mathis,[b#] Mengmeng Zhang,[a] Xuehang Wang,[b] Jianglan Shui,[c] Yury Gogotsi[b]* and Ming Xu[a]*

[a] *State Key Laboratory of Materials Processing and Die & Mold Technology, School of Materials Science and Engineering, Huazhong University of Science and Technology (HUST), Wuhan 430074, PR China.*

[b] *A. J. Drexel Nanomaterials Institute, and Department of Materials Science and Engineering, Drexel University, Philadelphia, Pennsylvania 19104, United States.*

[c] *School of Materials Science and Engineering, Beihang University, Beijing 100083, PR China.*

*e-mail: ming.xu@hust.edu.cn; gogotsi@drexel.edu

[#] These authors contributed equally

Supplementary Table 1 | Mass loading, thickness, density and resistivity of different MXene-knotted CNT composite electrodes.

**CNT ratio (%)**	**Mass loading ($mg\ cm^{-2}$)**	**Thickness (μm)**	**Density ($g\ cm^{-3}$)**	**Sheet resistance ($\Omega\ \square^{-1}$)**
0	2.38	8.1	2.93	0.15
5	2.35	9	2.61	0.5
9	2.2	9.2	2.39	3.5
17	2.1	9.8	2.14	6
34	1.8	10.7	1.68	10

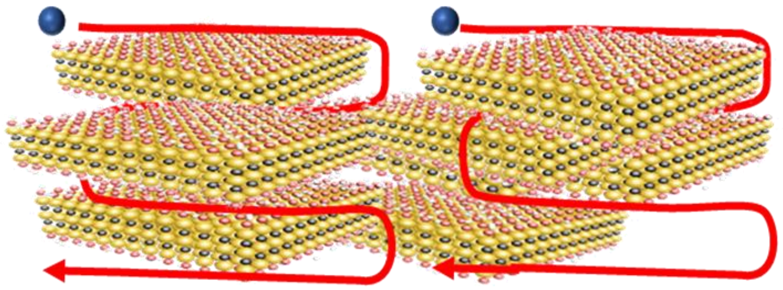

Supplementary Figure 1. Schematic of the MXene films with tortuous ions transport. Tortuous ions transport caused by restacking of the 2D nanosheets limits ion transport in the electrode.

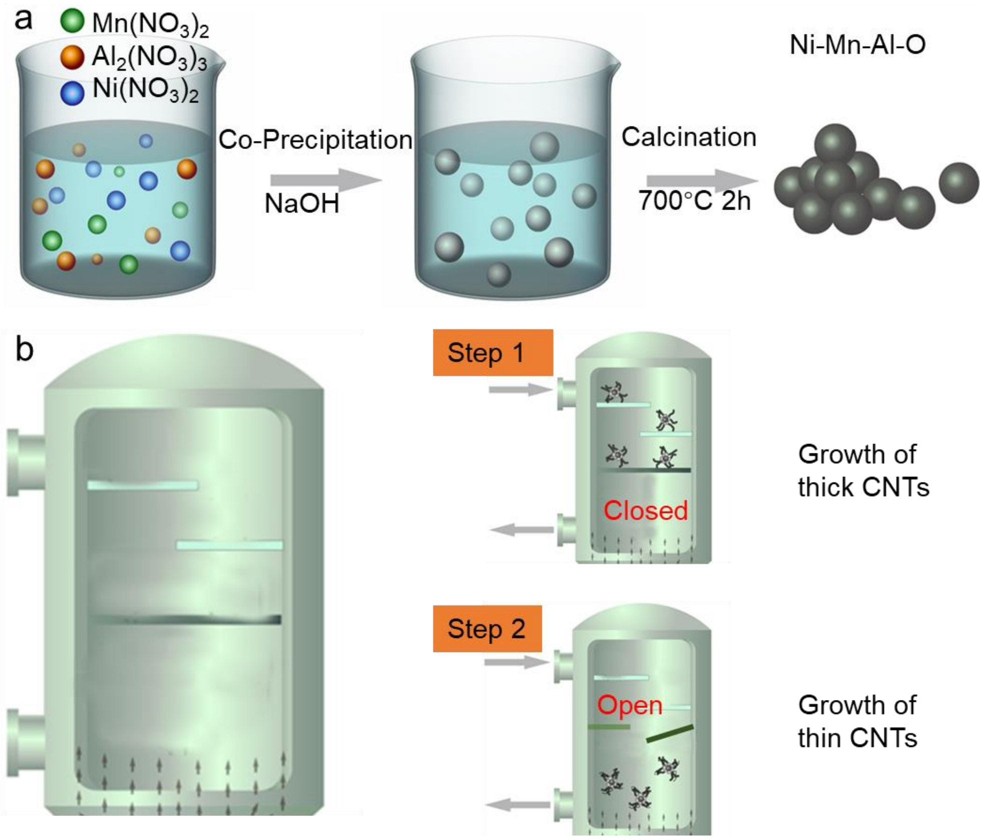

Supplementary Figure 2. Schematic for the synthesis of knotted CNTs. Schematic of (a) the synthesis of the catalyst and (b) the two-step growth of the knotted CNTs. The Ni-Mn-Al-O nanoparticles were prepared through the co-precipitation process followed by the calcination process. Then, the nanoparticles were loaded into the temperature shift two-stage fluidized bed for the growth of knotted CNTs.

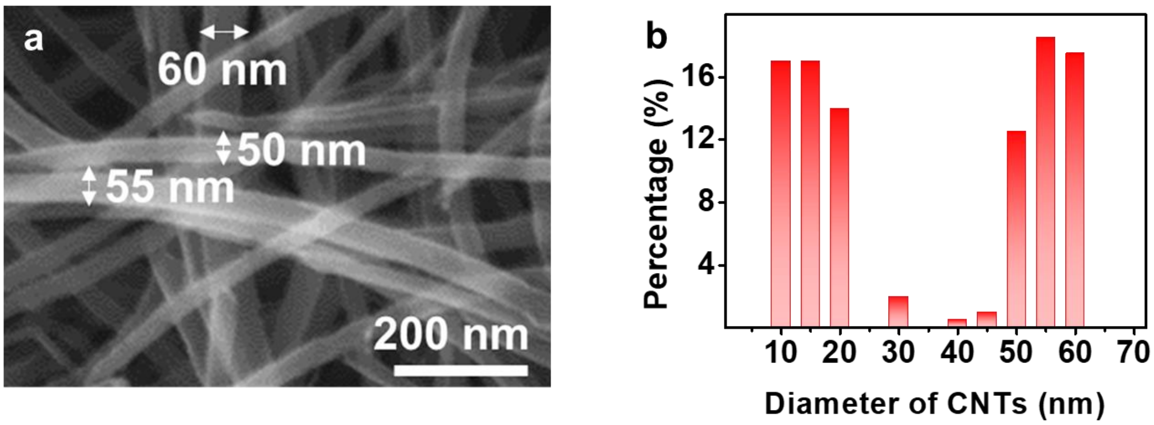

Supplementary Figure 3. Structural characterization of the knotted CNTs. (a) SEM image of the CNTs after first-step growth and (b) diameter distribution of the knotted CNTs after two-step growth. We could observe that the diameter of the CNTs was ~55 nm after first-step growth. After two-step growth, the knotted CNTs had a bimodal distribution, which were concentrated at ~15 nm and ~55 nm.

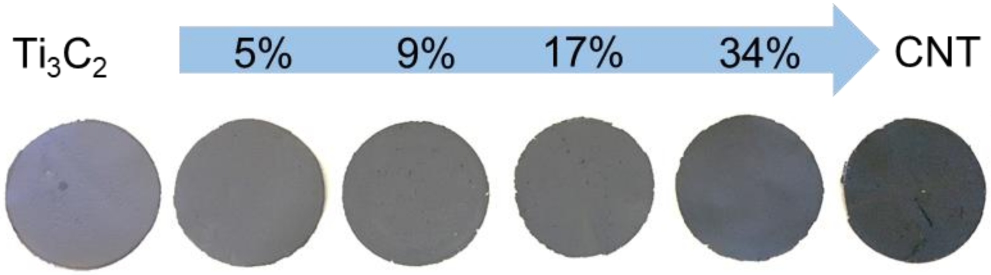

Supplementary Figure 4. Digital photos of different electrodes. All the MXene-knotted CNT composite electrodes were prepared at a diameter of ~4 cm. Besides, the color of the films experienced the change from purple to black with the increase of the CNT contents from 0 to 34%.

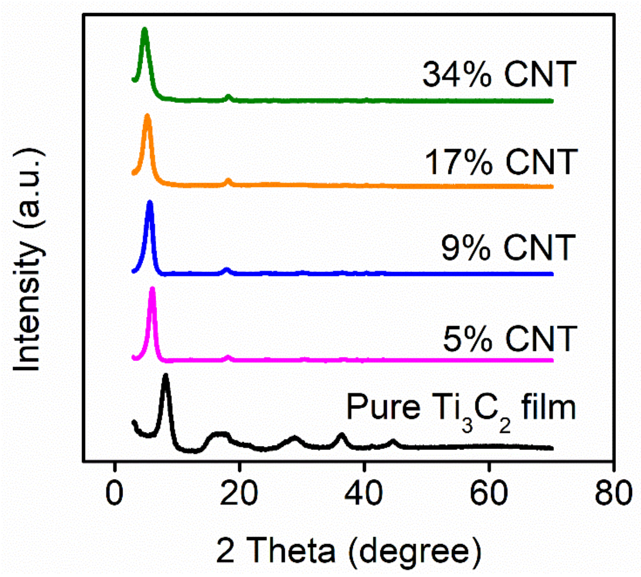

Supplementary Figure 5. XRD spectra of the MXene-knotted CNT composite electrodes with different CNT contents. The (002) peaks were located at 8.19°, 6.03°, 5.61°, 5.22° and 4.74° for the samples with the CNT contents from 0% to 34%, respectively. The corresponding interlayer spacings were 10.8 Å, 14.7 Å, 15.8 Å, 16.9 Å and 18.6 Å based on the calculation using the Bragg equation.[1, 2]

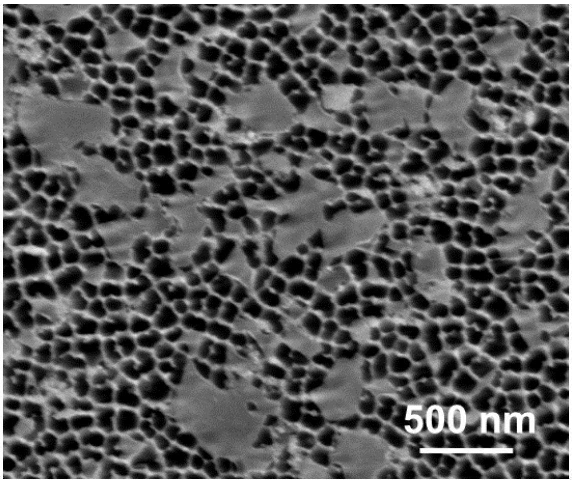

Supplementary Figure 6. The SEM image of the MXene flakes displayed the average flake size of ~250 nm.

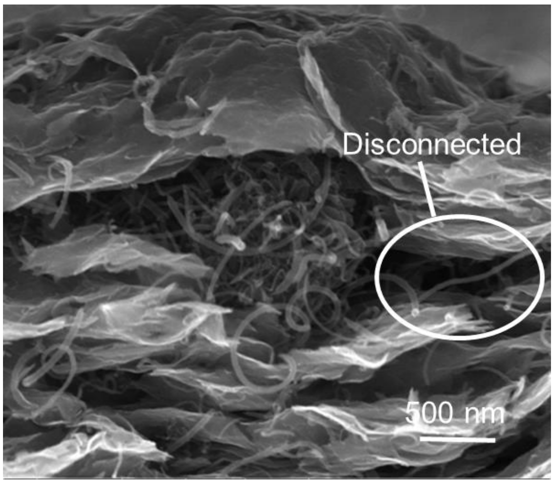

Supplementary Figure 7. SEM image of the MXene-knotted CNT composite electrodes with oversized CNT knots (~ 600 nm).

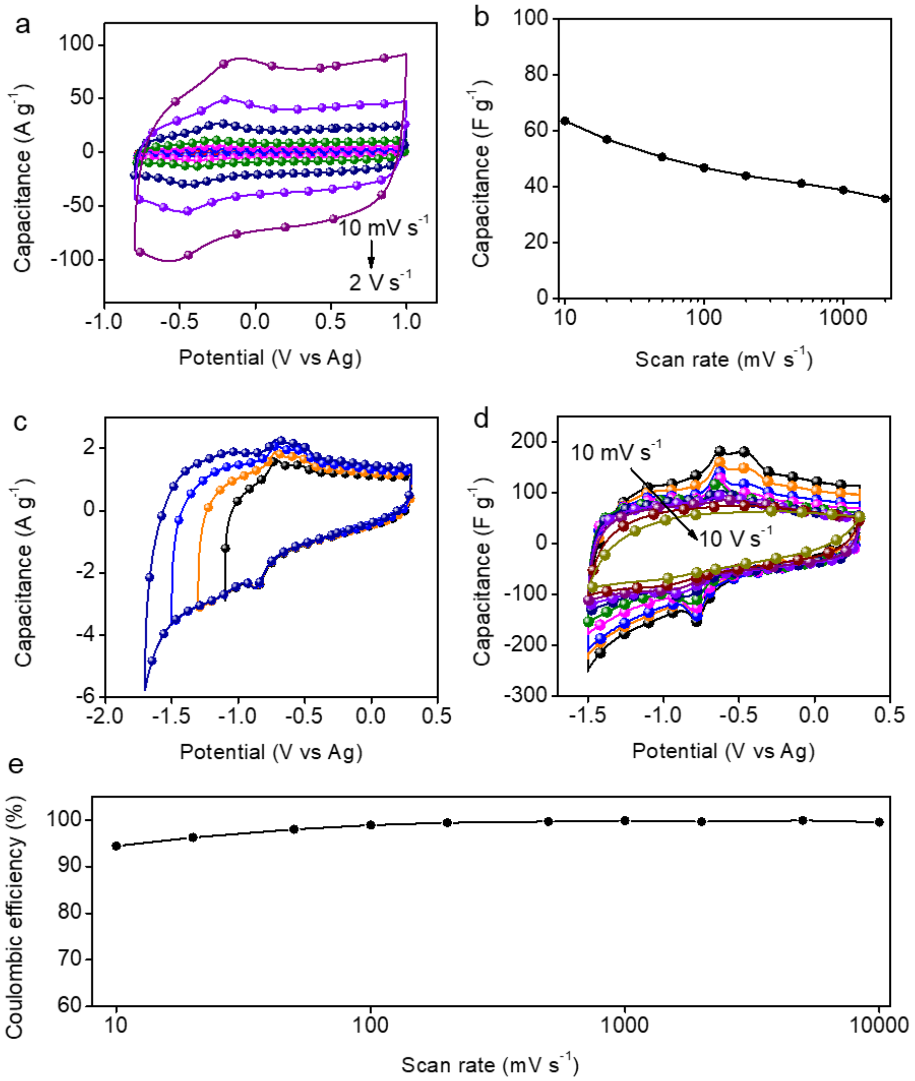

Supplementary Figure 8. Electrochemical performance of the MXene-knotted CNT composite electrode with the CNT content of 17% in different electrolytes. (a) Cyclic voltammogram (CV) curves tested at different scan rates in 1 M EMIM-TFSI ACN and (b) the corresponding capacitance at different scan rates. (c) CV curves tested at different potential window with a scan rate of 10 mV s^{-1} in an organic mixed electrolyte (EMIMTFSI: LiTFSI = 1: 1). (d) Plot of capacitance vs. potential derived from the CV curves from 0.3 V to -1.5 V at different scan rates and (e) the coulombic efficiencies at different scan rates in the organic mixed electrolyte. The coulombic efficiency increased from 94.4% to 99.4% at the scan rate increasing from 10 mV s^{-1} to 200 mV s^{-1} and maintained at ~100% at high scan rate from 200 mV s^{-1} to 10,000 mV s^{-1}.

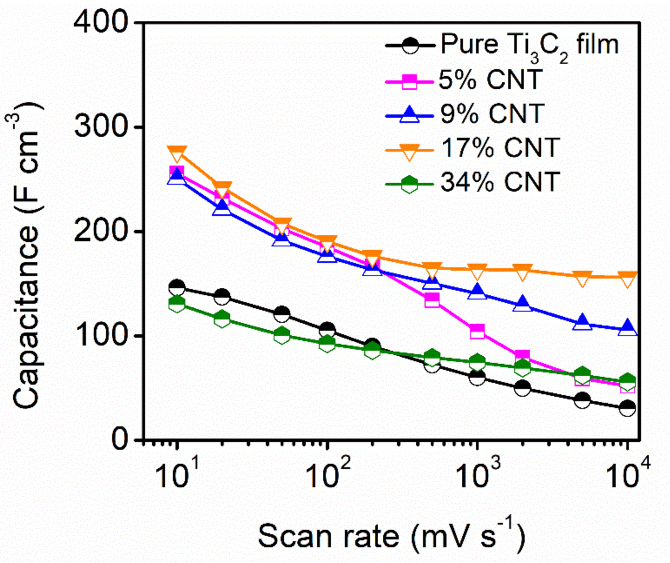

Supplementary Figure 9. Volume capacitance of the MXene-knotted CNT composite electrodes with different CNT contents. The densities of MXene and MXene-knotted CNT composite electrodes were shown in **Supplementary Table 1**.

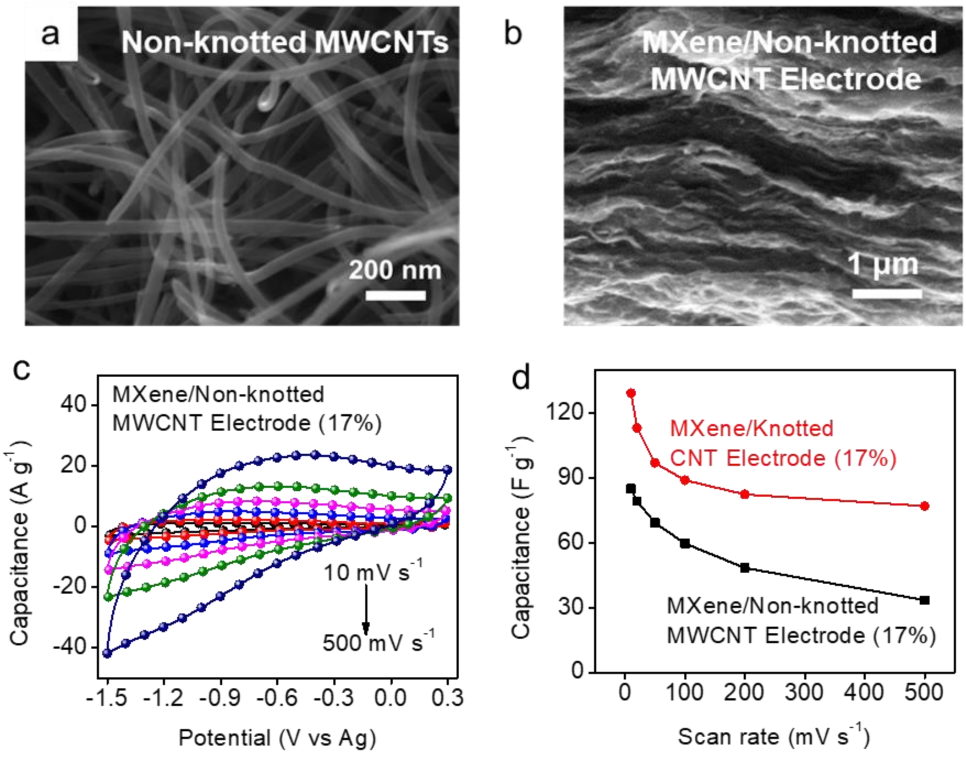

Supplementary Figure 10. Characterization and electrochemical performance of the MXene/non-knotted multi-wall carbon nanotube (MWCNT) electrode. SEM images of (a) non-knotted MWCNT and (b) MXene/non-knotted MWCNT electrode. (c) CV curves tested at different scan rates in 1 M EMIM-TFSI/Li-TFSI ACN electrolyte for MXene/non-knotted MWCNT electrode with the CNT content of 17%. (d) Comparison of the rate performance between MXene/knotted CNT electrode and MXene/non-knotted MWCNT electrode. **Supplementary Fig. 10a** displayed the MWCNT without knotted structure. **Supplementary Fig. 10b** showed the MXene/non-knotted MWCNT electrode with 2D stacked structure. The CVs of the MXene/non-knotted MWCNT electrode had the same potential window (1.8 V) as the MXene/knotted CNT electrode (**Supplementary Fig. 10c**). As seen in **Supplementary Fig. 10d**, the capacitance of the MXene/non-knotted MWCNT electrode decreased from 85 F g^{-1} to 33.5 F g^{-1} with the increasing of the scan rates from 10 mV s^{-1} to 500 mV s^{-1}. The capacitance retention of the MXene/non-knotted MWCNT electrode (39%) was much lower compared with that of the MXene/knotted CNT electrode (60%).

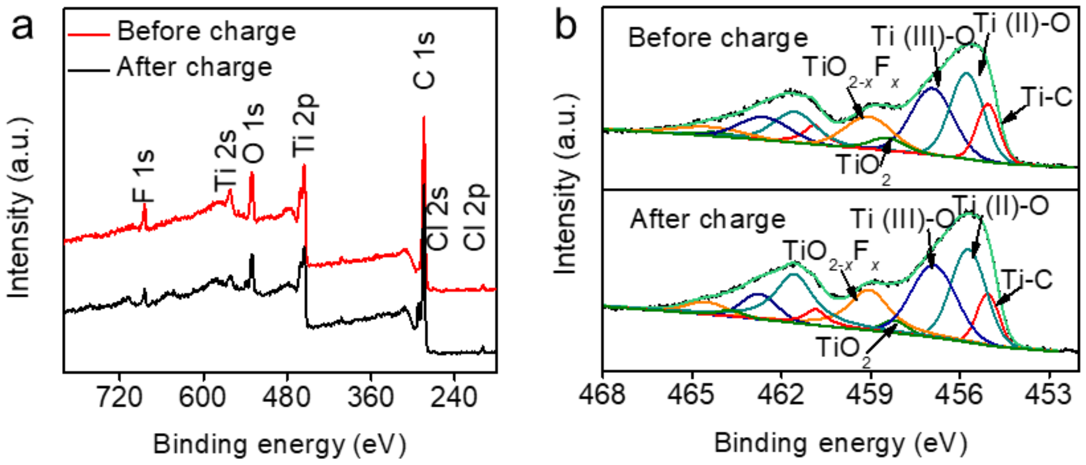

Supplementary Figure 11. XPS spectra of the MXene-knotted CNT composite electrode before and after charge. (a) General survey scan of the surface and (b) deconvolution of the Ti 2p peaks. From the general survey scan of the surface, the MXene composite electrodes have surface function groups of =O, -OH, -Cl and -F. The Ti $2p_{3/2}$ peak at 455.0 eV was associated with Ti bonded to C. The Ti $2p_{3/2}$ peaks at 455.8 eV and 457.1 eV were attributed to Ti atoms which were bonded to O. The Ti $2p_{3/2}$ peak at 458.6 eV was associated with TiO_2, while the Ti $2p_{3/2}$ peak at 459.2 eV corresponds to $TiO_{2-x}F_x$. No significant change of Ti 2p peaks w observed before and after charge, indicating that the electrochemical reactions do not significantly affect the surface chemistry of the MXene in this system.

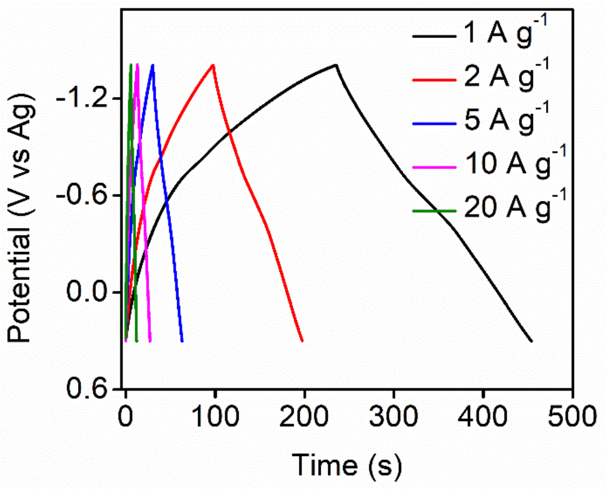

Supplementary Figure 12. Galvanostatic charge/discharge (GCD) curves at different current densities for MXene-knotted CNT composite electrode with the CNT content of 17%. The capacitance, which was calculated from the discharging curve, was ~128 F g^{-1} at 1 A g^{-1}.

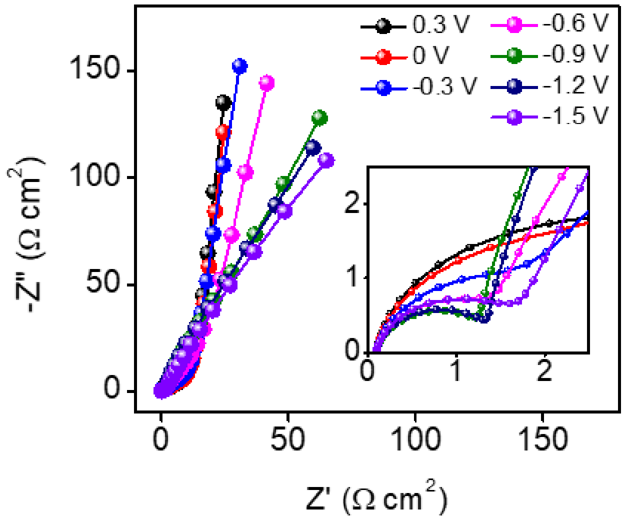

Supplementary Figure 13. Electrochemical impedance spectroscopy (EIS) data collected at different potentials for MXene-knotted CNT composite electrode with the CNT content of 17%. A slight increase of the 45-degree linear part (related to ion transport resistance) and a less steep slope of the Nyquist plot in the low-frequency range at -0.6 V were followed by a large increase of the 45-degree linear part at -0.9 V, -1.2 V and -1.5 V. This correlates with the appearance of redox peaks in the CVs.

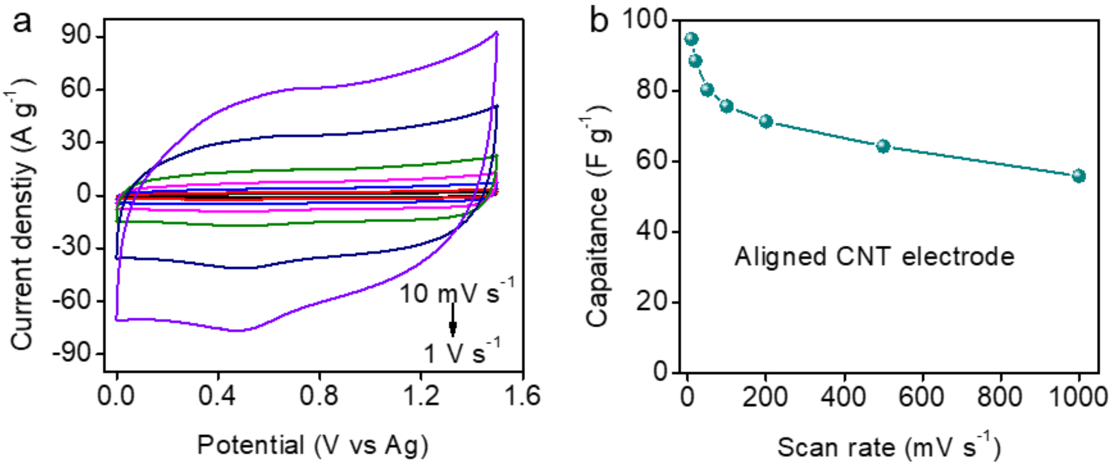

Supplementary Figure 14. Electrochemical performance of the aligned CNT electrodes. (a) CVs and (b) capacitance of the aligned CNT electrode at different scan rates. The aligned CNT electrode showed a capacitance of 94 F g^{-1} at 10 mV s^{-1} and a capacitance retention of 58% from 10 mV s^{-1} to 1 V s^{-1}. The capacitance of the asymmetric cell (C_{cell}) was obtained from the equation: $\frac{1}{C_{\text{cell}}} = \frac{1}{C_1} + \frac{1}{C_2}$ (C_1 and C_2 were the capacitance of each electrode). The capacitance of aligned CNT electrode (94 F g^{-1} at 10 mV s^{-1}) was lower compared to the MXene-knotted CNT composite electrode (~130 F g^{-1} at 10 mV s^{-1}). Since the capacitance of the cell was limited by the lower-capacitance electrode, the aggregate capacitance can be further improved by using the positive electrode possessing higher capacitance.

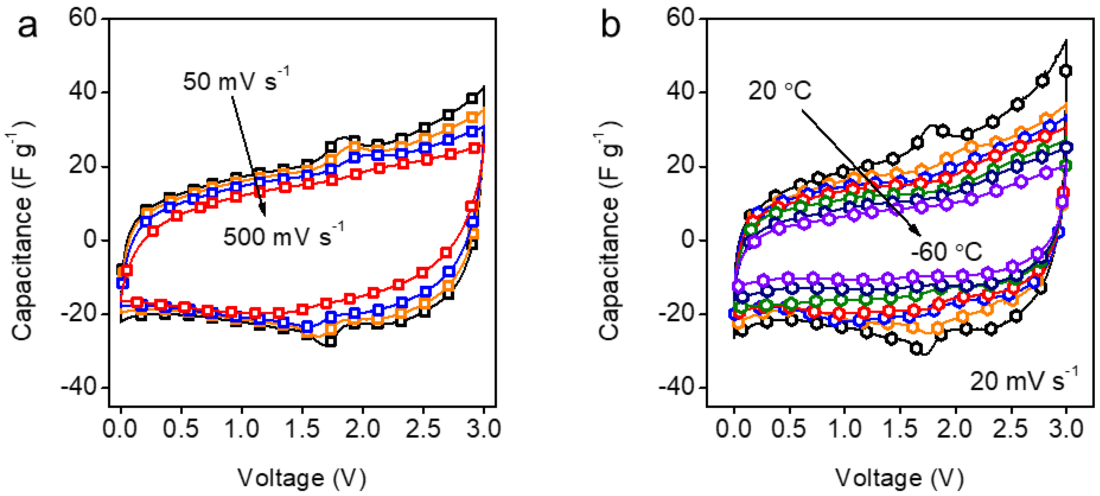

Supplementary Figure 15. Electrochemical performance of the asymmetric cells based on the MXene-knotted CNT composite electrode (CNT content of 17%). Plots of capacitance vs. voltage derived from (a) CVs at different scan rates and (b) CVs at different temperatures.

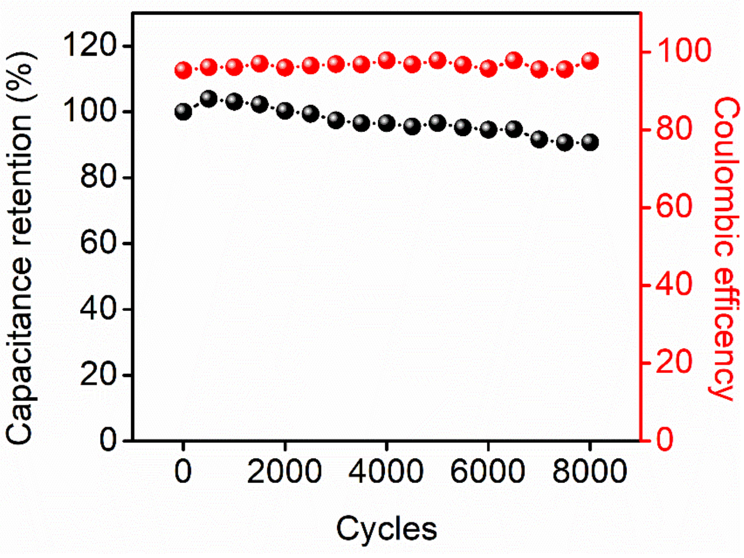

Supplementary Figure 16. Cycle performance of the asymmetric cell based on MXene-knotted CNT composite electrode with the CNT content of 17%. Capacitance retention of 90% was retained after 8,000 cycles. The coulombic efficiency of the cell was ~99%.

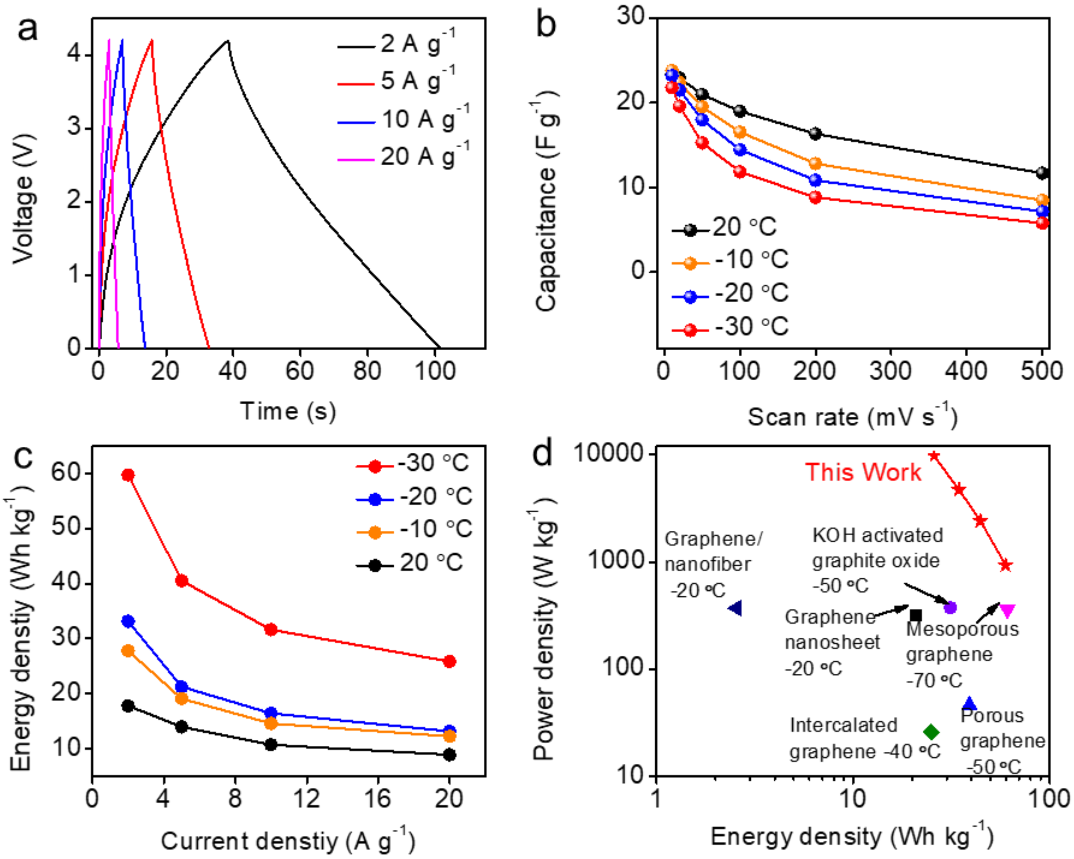

Supplementary Figure 17. Electrochemical performance of the asymmetric cells based on the MXene-knotted CNT composite electrode (CNT content of 17%) at low temperatures. (a) GCD curves at -30 °C, (b) rate performance at different temperatures, (c) energy density at different temperatures and (d) Ragone plots at -30 °C. At the lower temperature, we could pursue a larger voltage window. **Supplementary Fig. 17b** demonstrated that the capacitance at 10 mV s^{-1} remained almost unchanged with a capacitance of ~23 F g^{-1}. Due to the large voltage window of the cell, a high energy density of 59 Wh kg^{-1} at -30 °C was obtained (**Supplementary Fig. 17c**). **Supplementary Fig. 17d** compared the energy density and power density of the cells based on the 2D materials at low temperature. Our cell obtained much higher energy density and a high power density of 9.6 kW kg^{-1}, which was much higher than other 2D material-based supercapacitors at low temperature.[3-8]

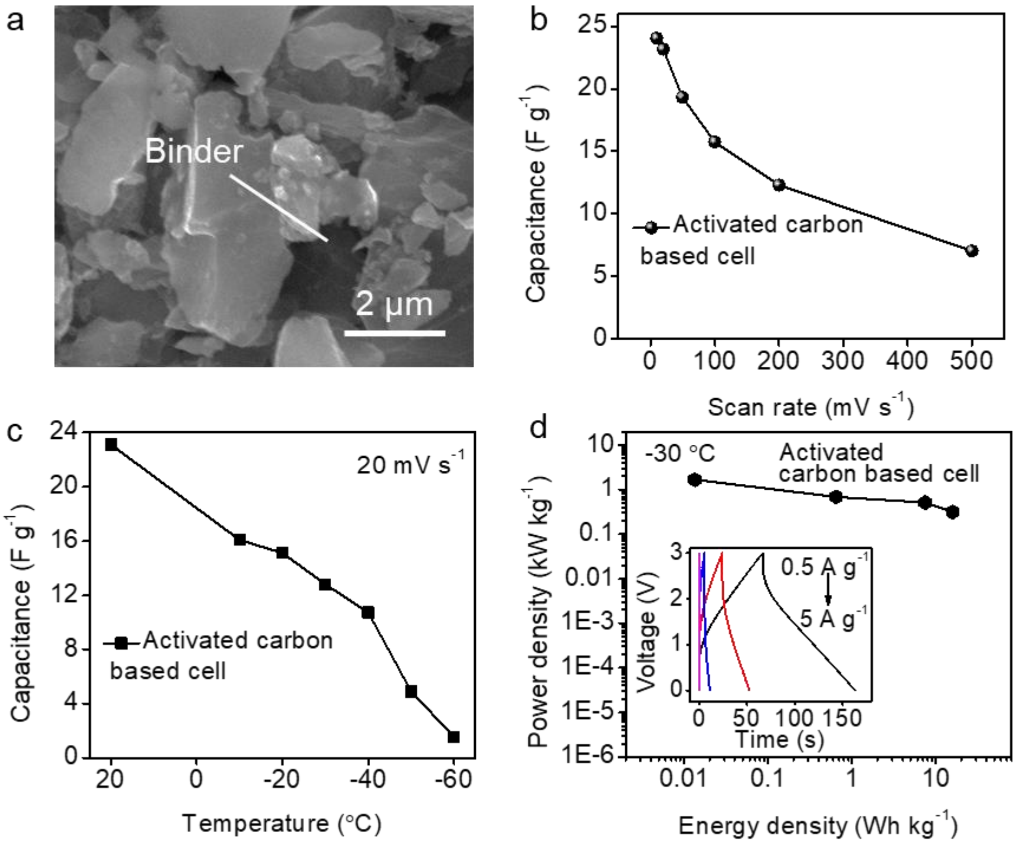

Supplementary Figure 18. Characterization and electrochemical performance the activated carbon (AC) electrode. (a) SEM image of the AC electrode. (b) Capacitance at different scan rates, (c) capacitance at different temperatures and (d) Ragone plots at -30 °C for AC based cell. Inset of **Supplementary Fig. 18d** showed the GCD curves of the AC based cell at -30 °C. The ACs showed particle sizes of 0.5-5 μm (**Supplementary Fig. 18a**). Due to the geometric discontinuity of AC particles, the binder (PTFE) and acetylene black had to be used to improve the conductivity of the electrode. The AC based symmetric cell showed a capacitance of 24 F g^{-1} at 10 mV s^{-1} and a capacitance retention of 29% from 10 mV s^{-1} to 500 mV s^{-1} (**Supplementary Fig. 18b**). In addition, the capacitance of the AC based symmetric cell decreased from 23 F g^{-1} to 1.5 F g^{-1} at 20 mV s^{-1} when the temperature decreased from 20 °C to -60 °C (**Supplementary Fig. 18c**). The symmetric cell displayed a voltage of 3 V and an energy density of 15 Wh kg^{-1} at -30 °C (**Supplementary Fig. 18d**) which was lower compared to the MXene-knotted CNT based cell (59 Wh kg^{-1}).

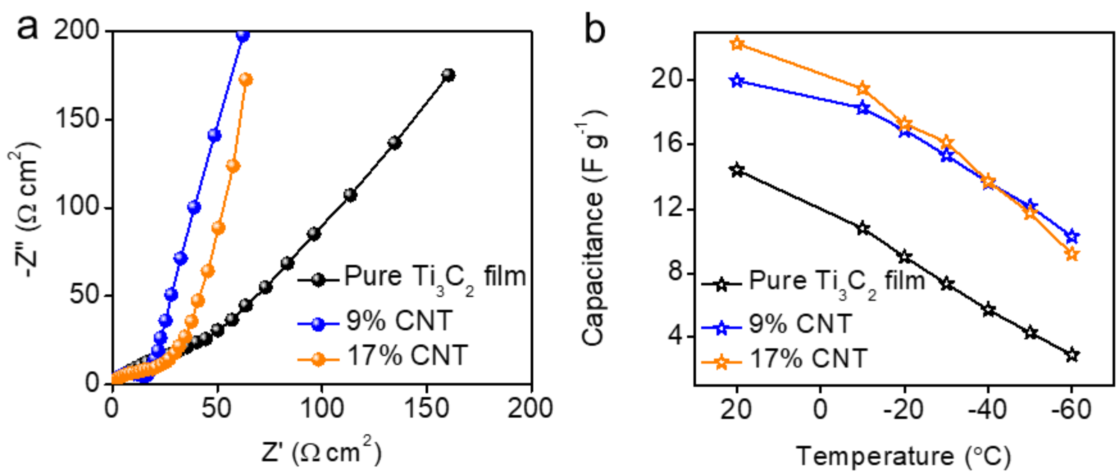

Supplementary Figure 19. Electrochemical performance of the asymmetric cells based on different electrodes. (a) EIS data and (b) capacitance at 20 mV s^{-1} from 20 °C to -60 °C of the asymmetric cells based on different electrodes. The Nyquist plots of asymmetric cells based on MXene-knotted CNT composite electrodes demonstrated nearly vertical lines normal to the real axis (Z') in the low frequency region while the Nyquist plot of the pure MXene film showed a nearly constant increase of both the imaginary and the real parts of the impedance. In addition, the charge transfer resistance (Rct), taken from the semicircles in the high to medium region, increased with the increase of the CNT contents from 9% to 17%. The increase of the Rct should be related with the enlarged interlayer spacing, which was supported by the XRD spectra. From **Supplementary Fig. 19b,** the asymmetric cells based on the MXene-knotted CNT composite electrode with CNT content of 17% had the highest capacitance of ~23 F g^{-1}.

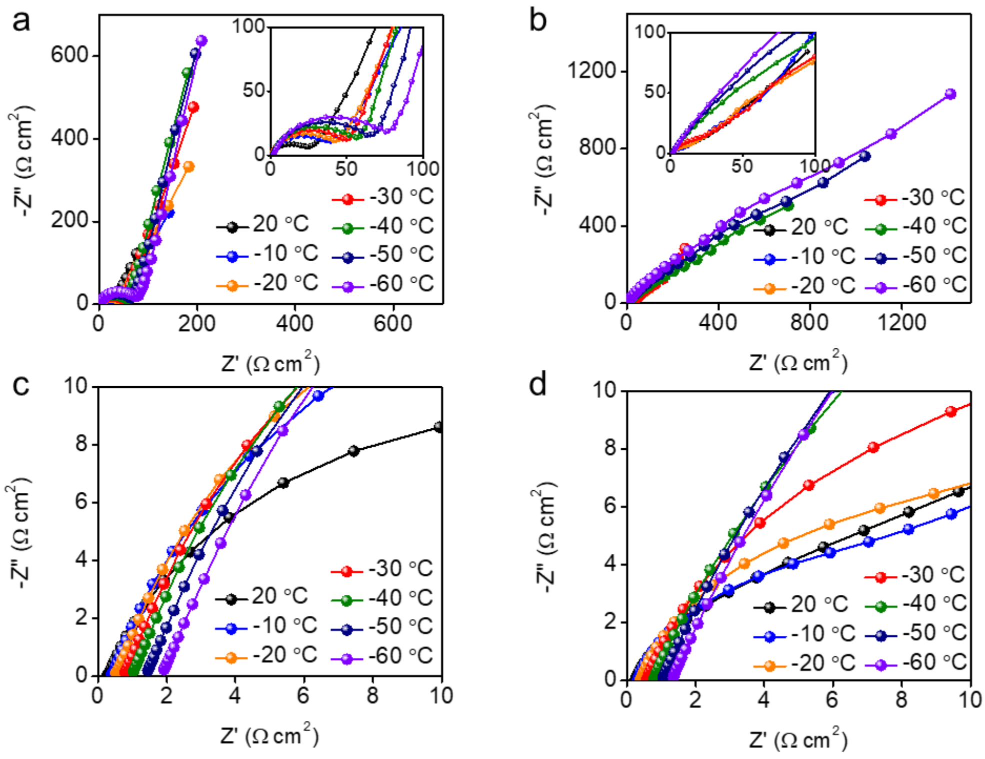

Supplementary Figure 20. EIS data of asymmetric cells based on different electrodes at different temperatures. (a) and (c) EIS data of asymmetric cells based on the MXene-knotted CNT composite electrode with the CNT content of 17% at different temperatures. (b) and (d) EIS data of asymmetric cells based on pure Ti_3C_2 film at different temperatures.

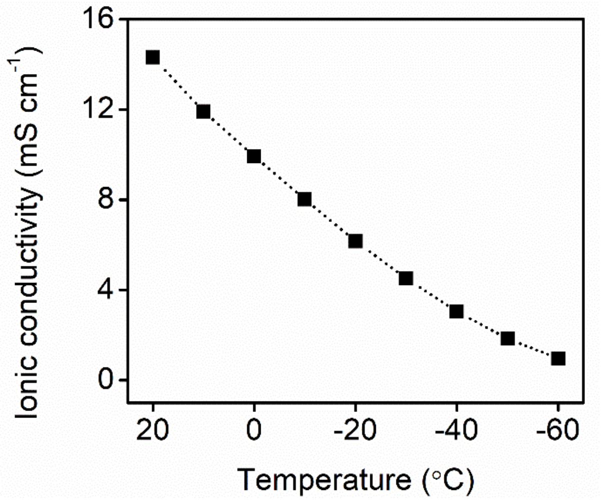

Supplementary Figure 21. Temperature dependence of the ionic conductivity for the electrolyte. The ionic conductivity of the electrolyte decreased from 14.31 mS cm^{-1} to 0.97 mS cm^{-1} when the temperature decreased from 20 °C to -60 °C.

Supplementary References

1. Lu, M. et al. Tent-pitching-inspired high-valence period 3-cation pre-intercalation excels for anode of 2D titanium carbide (MXene) with high Li storage capacity. *Energy Storage Mater.* **16**, 163-168 (2019).
2. Liu, Y. T. et al. Self-assembly of transition metal oxide nanostructures on MXene nanosheets for fast and stable lithium storage. *Adv. Mater.* **30**, 1707334 (2018).
3. Tsai, W.-Y. et al. Outstanding performance of activated graphene based supercapacitors in ionic liquid electrolyte from −50 to 80°C. *Nano Energy* **2**, 403-411 (2013).
4. Lin, Z., Taberna, P.L. & Simon, P. Graphene-Based Supercapacitors Using Eutectic Ionic Liquid Mixture Electrolyte. *Electrochim. Acta* **206**, 446-451 (2016).
5. Liu, W., Yan, X., Lang, J. & Xue, Q. Effects of concentration and temperature of $EMIMBF_4$/acetonitrile electrolyte on the supercapacitive behavior of graphene nanosheets. *J. Mater. Chem.* **22**, 8853 (2012).
6. Zhou, Y. et al. High performance supercapacitor under extremely low environmental temperature. *RSC Adv.* **5**, 71699-71703 (2015).
7. Tian, J. et al. $EMIMBF_4$–GBL binary electrolyte working at −70 °C and 3.7 V for a high performance graphene-based capacitor. *J. Mater. Chem. A* **6**, 3593-3601 (2018).
8. Vellacheri, R. et al. High performance supercapacitor for efficient energy storage under extreme environmental temperatures. *Nano Energy* **8**, 231-237 (2014).
