# Supplementary material for: Maximizing ion accessibility in MXene-knotted carbon nanotube composite electrodes for high-rate electrochemical energy storage
Source: Nat Commun. 2020 Dec 2;11:6160. doi: 10.1038/s41467-020-19992-3 (PMC7710708; doi:10.1038/s41467-020-19992-3)
Supplement: Supplementary file 2 — Peer Review File [file 41467_2020_19992_MOESM2_ESM.pdf]

Reviewer #1 (Remarks to the Author):

In the manuscript entitled “Maximizing ion accessibility in MXene-knotted carbon nanotube composite electrodes for high-rate electrochemical energy storage”, a MXene-knotted carbon nanotube composite was fabricated. The author illustrated how the architecture of an electrode can lead to enhanced rate performance inorganic electrolytes. This is an interesting and systematic work that is worthy of publishing in Nature Communications. However, there are still several points found in the manuscript requiring further elucidations after my reading in depth.

1. Similar work about the electrochemical energy storage of MXene-CNT composites has been reported previously [J. Power Sources 306, 510-515; Nano Energy 26, 513-523 (2016).]. The authors should emphasize the differences between the present work and the previous work.
2. The authors emphasized the knotted structure on the performance of the MXene-CNTs composites, while the performance of MXene-CNTs composites without knotted structure is unknown. In order to support the authors’ statement, a comparison should be made.
3. How about the layer spacing of the MXene knotted CNT composite with different CNT content?
4. In section 2, the authors claimed: “Decreases in capacitance and capacitance retention occurred when the CNT content was increased to 34%, which is likely due to the larger internal resistance of the 34% CNT electrode due to the discontinuous MXene network.” As far as I know, CNT also has very high electrical conductivity, which is inconsistent with the authors’ statement. If the authors insist this statement, they should measure the conductivity of these samples to provide supporting.
5. The author claimed that the layer spacing of MXene is the key fact that influence the ion accessibility. Does the layer spacing the larger the better? If not, what is the best value of the layer spacing?

Reviewer #2 (Remarks to the Author):

This is a very interesting paper that clearly demonstrates the effect of decorating MXenes with knotted CNTs on the rate response in organic electrolytes even at low temperatures. The results are novel and of interest mainly for the supercapacitor community. However, despite many data are reported and deeply discussed, I feel that the paper, at present, does not meet the high standard required for publication on Nature Communication for the reasons listed below.

1. In the Introduction and in the Discussion sections, the authors never refer to electrode thickness. Designing materials that keep the same performance when the mass loading is increased from 1 to 10 mg/cm2 is one of the biggest challenges that have to be addressed both for supercapacitors and battery development. It is extremely important to indicate the electrode mass loading because it enables to clearly compare the performance of novel materials with state of art systems (J. Li, J. Power Sources, 452 (2020) 227824). This information for MXenes-knotted CNTs is not reported nor commented, so that results appear not very outstanding.

2. The specific energy and power values have been obtained from cyclic voltammetry measurements. This can be misleading. Indeed, energy and power should be evaluated by the galvanostatic discharge curves. The reason is that energy and power strongly depend on current. The Nyquist plots reported in Figure 15 show that the cells feature more than 200 ohm cm2. This means that practical current densities for the cells assembled with MXenes-knotted CNTs cannot be higher than few mA/cm2, that is a low value for supercapacitors.
The authors report galvanostatic tests at 10 A/g, but given that the mass loading is not given, it is not possible to get the current density. Therefore, the claim about impressive energy density and power is not very convincing.

3. The ionic conductivity of the electrolyte at the different temperatures is not reported. This information is needed to better understand the evolution of the Nyquist plots with temperature.

4. The electrochemical characterization of knotted CNTs - MXenes is presented only in the negative potential domain. I understand that this material cannot be used as positive electrode. This information is not clearly given. Furthermore, an aligned carbon nanotube electrode is used as the positive electrode, but electrochemical data (mass, specific capacitance, voltammetric reponse,…) on this electrode are not reported. These data are needed to understand the real contribution of knotted CNTs – Mxenes to the full cell performance

5. While the capacitance retention with scan rate and temperature of the materials and the full cells are interesting, the electrode and cell specific capacitance values are not outstanding. Indeed, the authors report about a cell capacitance of 20F/g, but commercial supercapacitors typically feature 20-25F/g. A comparison with commercial high surface area carbons tested in the same electrolyte should be reported in order to better highlight knotted CNTs – Mxenes performance.

6. Additional data (like XPS) on the surface chemistry of the knotted CNTs - MXenes Should be reported to exclude any pseudocapacitive contribution that colud be related to surface moieiteis.

Minor comments:
Page 2. "organic electrolytes have larger charge storing ions ". I suggest to change it with "organic electrolytes have larger ions" and to add a comment about different solvation shells of ions in organic solvents and water.
-Figure 4b. It is not correct to report the y-axis in terms of specific capacitance because specific capacitance has not negative values. Voltammetries should be reported in terms of current density or specific current.
-Page 19. Energy and power should be calculated from the galvanostatic tests. In any case, these values should refer to the discharge. In case of the voltammetric analyses of the full cell, they should be obtained from the cathodic scan (negative currents) and not from the anodic scan.

Reviewer #3 (Remarks to the Author):

The authors have studied and reported the use of a knotted CNT for disrupting the alignment of the Ti_3C_2 flakes, leading to increased ion transport pathways and ion accessibility. The work is quite interesting as it also deals with storage devices working at sub-zero temperatures. However, the following clarifications with some minor corrections are necessary for the manuscript to be considered for publication.
1. The authors are advised to perform the spell check thoroughly. Ex: 'remaines' in introduction. In addition, there is a mixture of past and present tenses at some parts.
2. Does the growth of thick and thin CNTs depends on the temperature shift or with the use of different sized catalysts? Do both have an effect? Kindly clarify.
3. The authors have mentioned that by varying the size of the catalyst, different sized CNTs were obtained. How have the authors varied the size of the catalyst and how is it confirmed? The methods section do not discuss on obtaining various sized catalysts.
4. The authors could discuss on the mechanical stability of the knotted CNTs at large cycles.
5. The size of the CNT knots is mentioned to be 200 ± 20 nm and that of Ti_3C_2 flakes as ~250 nm. How have the authors confirmed the exact size of these particles?
6. Details on the aligned CNT used as positive electrode in ASC could be discussed.
7. The authors could compare the electrochemical performance of MXene with the MXene-CNT composite.

Reviewer #1 (Remarks to the Author):

In the manuscript entitled "Maximizing ion accessibility in MXene-knotted carbon nanotube composite electrodes for high-rate electrochemical energy storage", a MXene-knotted carbon nanotube composite was fabricated. The author illustrated how the architecture of an electrode can lead to enhanced rate performance inorganic electrolytes. This is an interesting and systematic work that is worthy of publishing in Nature Communications. However, there are still several points found in the manuscript requiring further elucidations after my reading in depth.

Response: Thank you for your time and valuable comments on our work, please see our detailed response below.

1. Similar work about the electrochemical energy storage of MXene-CNT composites has been reported previously [J. Power Sources 306, 510-515; Nano Energy 26, 513-523 (2016).]. The authors should emphasize the differences between the present work and the previous work.

Response: Please see Table R1 for a comparison between our work and previous related studies.

Table R1. Comparison between our work and related literatures

		Our work	**Ref 1**: Dall'Agnese, Y. et al. J. Power Sources 306, 510-515 (2016).	**Ref 2**: Xie, X. et al. Nano Energy 26, 513-523 (2016)
Electrode structure		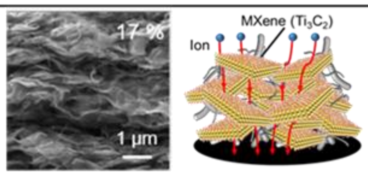 3D electrolyte-accessible architecture with efficient ion transportation by dramatically reducing the tortuosity of the ion transport pathways (Figure 1b and Figure 2g)	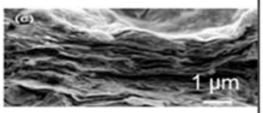 CNTs are homogeneously spread between the Ti_3C_2 layers (**tortuous ion transport**)	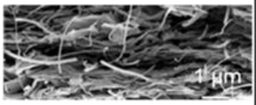 The composite film has an open sheet arrangement and CNTs are sandwiched between Ti_3C_2 (**tortuous ion transport**)
Mechanism		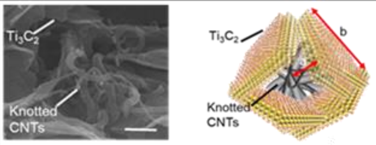 Size match of the Large knots and Ti_3C_2 resulted in the misalignment of the 2D Ti_3C_2 flakes, leading to enhanced accessibility and improved transport of charge storing ions through the electrode (Figure 2e and f).	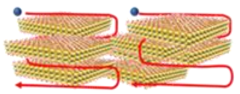<br>Open 2D structure of the material and high accessibility of the surface to ions (**tortuous ion transport**)	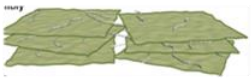<br>Larger interlayer space and improved ion accessibility (**tortuous ion transport**)
**Property**	Rate Capability	130 F/g (276 F/cm^3) at 10 mV/s<br><br>10 mV/s to 10 V/s with a capacitance retention of 56%	85 F/g (245 F/cm^3) at 2 mV/s<br><br>2 mV/s to 100 mV/s (**Low scan rates**)	75 F/g (175 F/cm^3) at 10 mV/s<br><br>0.5 mV/s to 100 mV/s (**Low scan rates**)
	Low-Temperature performance	A high capacitance retention of ~55% was obtained over the temperature range of 20 °C to -60 °C at 20 mV/s.	-	-
	Voltage	4.2 V voltage window without any parasitic reactions or losses in capacitive behavior	1.8 V	3 V
	Energy density and power density	An energy density of 59 Wh/kg and a power density of 9.6 kW/kg were obtained at -30 °C	-	-

The paper (*J. Power Sources* 306, 510-515 (2016) as Ref. 16 in the manuscript) is about sandwich-like

MXene/multiwall CNT (MWCNT) composite electrodes produced by directly mixing a colloidal solution of delaminated Ti_3C_2 with common MWCNTs. Although a 2D (not 3D) stacked structural electrode was demonstrated that had increased interlayer spacing, the ion transport in the electrode remained tortuous and the increases in ion accessibility were limited. As a result, the capacitance of 85 F/g (245 F/cm^3) at 2 mV/s was presented with the rate performance shown only from 2 mV/s to 100 mV/s.

Another work (*Nano Energy* 26, 513-523 (2016) as Ref. 13 in the manuscript) demonstrated a MXene/MWCNT composite electrode produced through self-assembly of negatively charged $Ti_3C_2T_x$ MXene flakes and positively charged CNTs as spacers. The electrode structure is similar with the J. Power Sources paper. Thus, the ion transport in their electrodes was also tortuous and with limited improvements in ion accessibility. As a result, the capacitance of 75 F/g (175 F/cm^3) at 10 mV/s was presented with the rate performance only from 0.5 mV/s to 100 mV/s.

In our work, we have fabricated 3D electrolyte-accessible electrodes by misaligning the MXene flakes through the knotted CNTs, which presents a conceptually new electrode structure. This structure enables both ion transport pathways with significantly reduced tortuosity and much enhanced ion accessibility. Compared to the 2D stacked electrode structures in the papers referred in the review comment, the tortuosity of ion- transport pathways of our 3D electrolyte-accessible electrode is significantly reduced.

We have added the sentences in the following to the introduction of our manuscript to emphasize these differences: "For example, Dall'Agnese *et al.* used multiwall carbon nanotubes (MWCNTs) as the spacers to separate MXene sheets and reported the capacitances ranging from 85 F/g (245 F/cm^3) at 2 mV/s to 63 F/g (181 F/cm^3) at 100 mV/s.[16] Another study reported MXene/MWCNT composite electrodes prepared by self-assembly of negatively charged, metallic $Ti_3C_2T_x$ MXene flakes and positively charged CNTs as spacers. The capacitance of 75 F/g (175 F/cm^3) at 10 mV/s was achieved, with the rate performance from 0.5 mV/s to 100 mV/s.[13] *Although the MXene/MWCNT composite electrodes produced by these approaches appear to have a larger interlayer spacing for their improved ion accessibility compared to unmodified MXenes, the scanning rate has never been achieved higher than 100 mV/s and only room-temperature properties have been reported. Even though these methods increase the spacing between the layers of the 2D electrode materials (e.g. MXene), the stacking problem persists that the 2D layers remain horizontally stacked. Therefore, the ion transport in the electrode is very tortuous and slow in the direction normal to the electrode surface, which would be an obstacle for high-rate performance and low-temperature operation.*" (Pg. 3 Line 21& Pg. 4, Lines 1-7)

2. The authors emphasized the knotted structure on the performance of the MXene-CNTs composites, while the performance of MXene-CNTs composites without knotted structure is unknown. In order to support the authors' statement, a comparison should be made.

Response: According to your suggestion, we have assembled the MXene composite electrode with the non-knotted CNTs made from commercially available ones and compared the performance with our electrodes with knotted CNTs as below.

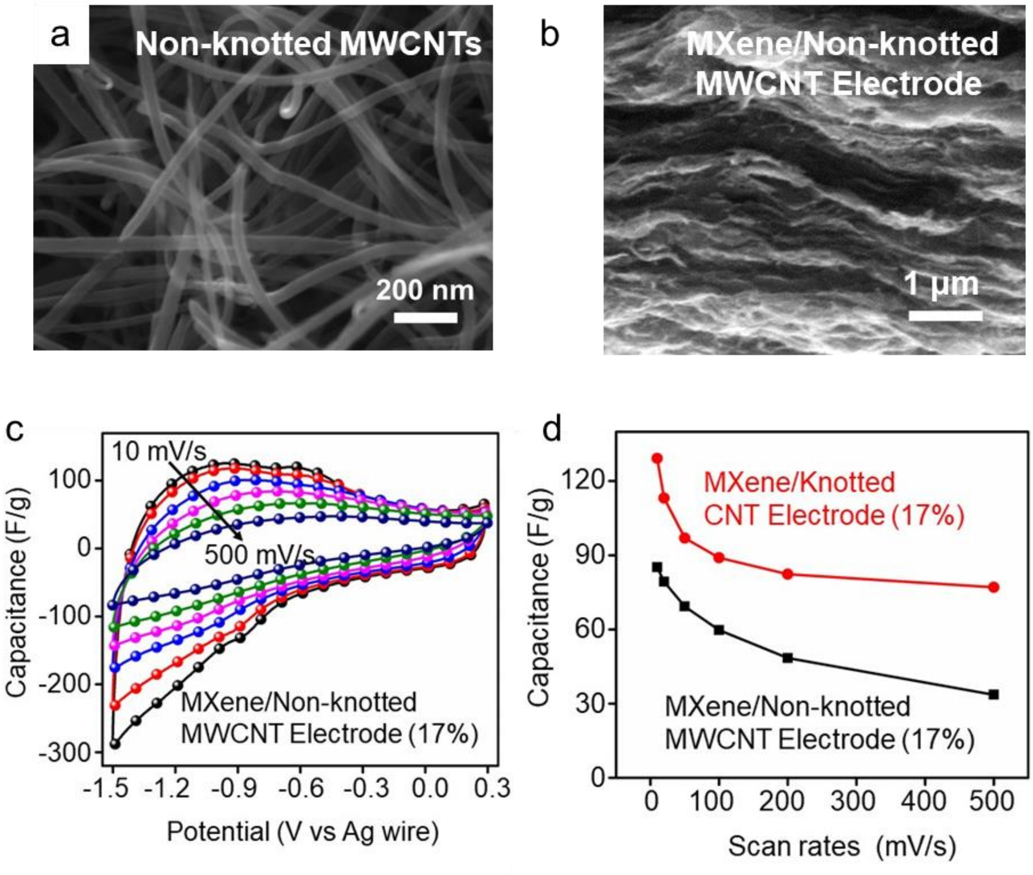

Figure R-1. SEM images of (a) non-knotted multi-wall carbon nanotube (MWCNT) and (b) MXene/non-knotted MWCNT electrode. (c) CV curves tested at different scan rates in 1 M EMIM-TFSI/Li-TFSI ACN electrolyte for MXene/non-knotted MWCNT electrode with a CNT content of 17%. (d) Comparison of the rate performance between MXene/knotted CNT electrode and MXene/non-knotted MWCNT electrode.

Figure R-1a showed the straight tube structure of the commercially available non-knotted MWCNTs with a uniform diameter of ~35 nm. Figure R-1b showed a 2D stacked structure of the composite electrode by mixing MXene and the non-knotted MWCNTs, which was very different from our 3D electrolyte-accessible electrodes structure (17% CNTs electrode in **Fig. 2g**). As shown in Fig. R-1c&d, the capacitance of the MXene-non-knotted MWCNT electrode decreased from 85 F/g to 33.5 F/g with the increase of the scan rates from 10 mV/s to 500 mV/s. The capacitance retention was only 39% from 10 mV/s to 500 mV/s which was much lower than that of the MXene-knotted CNT composite electrode (56% from 10 mV/s to 10 V/s).

We have already added Figure R-1 in the supporting information as **Supplementary Fig. 10** and added the description in Pg.12 as "*Moreover, we prepared the composite electrode by mixing MXene and the commercially available non-knotted MWCNTs for comparison (**Supplementary Fig. 10**). As shown by **Supplementary Fig. 10a-b,** the non-knotted MWCNTs possessed a uniform diameter of ~35 nm and the electrode displayed a 2D stacked structure, which was very different from the 3D electrolyte-accessible electrodes structure formed by our knotted CNTs. The MXene/non-knotted MWCNT electrode only showed a capacitance retention of 39% from 10 mV/s to 500 mV/s (**Supplementary Fig. 10c-d**). The results indicated that the ion transport in the MXene/non-knotted MWCNT electrode remained tortuous and the increases in ion accessibility were still limited even when the interlayer spacing of the electrode was increased compared to the pure MXene. It is clear that the construction of a 3D electrolyte-accessible electrode structure through the use of specially synthesized knotted CNTs has a significant effect on both reducing the tortuosity of ion transport and increasing ion accessibility.*"

3. How about the layer spacing of the MXene knotted CNT composite with different CNT content?

Response: We have tested the XRD spectra of the different MXene-knotted CNT composites and the data is presented in **Supplementary Fig 5**. We have also added the relevant analysis in the paper (Pg. 9, Lines 12-14) as "*The (002) peaks were located at 8.19°, 6.03°, 5.61°, 5.22° and 4.74° for the samples with CNT content from 0% to 34%, respectively. The corresponding interlayer spacings calculated using the Bragg equation were 10.8 Å, 14.7 Å, 15.8 Å, 16.9 Å and 18.6 Å.*[27, 28]".

4. In section 2, the authors claimed: "Decreases in capacitance and capacitance retention occurred when the CNT content was increased to 34%, which is likely due to the larger internal resistance of the 34% CNT electrode due to the discontinuous MXene network." As far as I know, CNT also has very high electrical conductivity, which is inconsistent with the authors' statement. If the authors insist this statement, they should measure the conductivity of these samples to provide supporting.

Response: Although CNTs have high electrical conductivity, physical contact between the conductive materials in the electrode is also very important for the overall conductivity of the electrode. Furthermore, the conductivity of CNTs is lower than MXene, which has conductivity values exceeding 15,000 S/cm (Adv. Mater. 32, 202001093 (2020)). Thus, having a continuous MXene structure is highly important for the bulk conductivity of the MXene composite electrodes. We have tested the conductivity of the different MXene knotted CNT composite films and shown that the sheet resistance increases from 0.15 Ω/□ to 10 Ω/□ as the CNT content increases. The conductivity of the electrodes has been added in the ***Supplementary Table 1*** and the description has been added in Pg. 11, lines 1-2 as "Incorporating higher amounts of knotted CNTs likely led to separation of the MXene and CNT phases within the electrode structure due to the significant difference in the densities of the CNTs and Ti_3C_2, which would affect the conductivity of the electrodes. *The sheet resistance conductivity of different MXene-knotted CNT composite films increased from 0.15 Ω/□ to 10 Ω/□ with the increasing of the CNT contents from 0% to 34% (**Supplementary Table 1**).*"

5. The author claimed that the layer spacing of MXene is the key fact that influence the ion accessibility. Does the layer spacing the larger the better? If not, what is the best value of the layer spacing?

Response: From the XRD spectra (**Supplementary Fig. 5)**, the interlayer spacing of the electrodes increases as the CNT content increases. However, the best electrochemical performance was realized by the electrode with CNT content of 17%. Therefore, simply expanding the interlayer spacing is not the only factor that influences electrochemical performance.

It would be more accurate to say that creating an electrolyte accessible electrode structure is crucial for optimizing the performance. The electrode in our study with the best performance (17% CNT content) had a 3D electrolyte-accessible architecture that was the result of the misalignment of the MXene flakes by the knotted CNTs. While this process does increase the interlayer spacing, our study presents a new electrode structure concept that considers both ion transport pathways with reduced tortuosity (disrupted MXene flake stacking) and enhanced ion accessibility (open structure and increased interlayer spacing). Compared to the 2D stacked electrode structures in previous reports [*J. Power Sources* 306, 510-515 (2016); *Nano Energy* 26, 513-523 (2016).], the tortuosity of the ion transport pathways in our electrodes has been significantly reduced. Thus, we should consider both the layer spacing and the ion transport pathways when designing the electrode, which we have emphasized in Pg. 9, lines 16-20 as "*The entirety of the electrode structure was composed of CNT knots encased by Ti_3C_2 flakes, exhibiting a 3-D architecture with both increased interlayer spacing and misaligned MXene flakes. Compared to 2D stacked electrode structures that only had enlarged interlayer spacing, our 3-D electrode structure provided increased open space for enhanced accessibility and reduced the tortuosity of the ion transport pathways to improve the transport of charge storing ions through the electrode.*"

Reviewer #2 (Remarks to the Author):

This is a very interesting paper that clearly demonstrates the effect of decorating MXenes with knotted CNTs on the rate response in organic electrolytes even at low temperatures. The results are novel and of interest mainly for the supercapacitor community. However, despite many data are reported and deeply discussed, I feel that the paper, at present, does not meet the high standard required for publication on Nature Communication for the reasons listed below.

Response: Thank you for your time and valuable comments on our work, please see our detailed response below.

1. In the Introduction and in the Discussion sections, the authors never refer to electrode thickness. Designing materials that keep the same performance when the mass loading is increased from 1 to 10 mg/cm^2 is one of the biggest challenges that have to be addressed both for supercapacitors and battery development. It is extremely important to indicate the electrode mass loading because it enables to clearly compare the performance of novel materials with state of art systems (J. Li, J. Power Sources, 452 (2020) 227824). This information for MXenes-knotted CNTs is not reported nor commented, so that results appear not very outstanding.

Response: We absolutely agree with the reviewer and the opinion of J. Li, *J. Power Sources*, 452 (2020) 227824 that the mass loading and the thickness of the electrode are very important to compare the performance of novel materials. We actually provided the mass loading, density, and the thicknesses of the electrodes in **Supplementary Table 1**. As can be seen, the thickness of the electrodes increased from 8.1 μm to 10.7 μm and the mass loading of the electrodes decreased from 2.38 mg/cm^2 to 1.8 mg/cm^2 as the CNT content increased.

We are very sorry that the original manuscript probably didn't make this clear. We have now added the following line to emphasize the mass loading and thicknesses of the electrodes in the manuscript to avoid future misunderstandings: "*The thicknesses of the electrodes were controlled to be approximately 10 μm and all the electrodes displayed a mass loading higher than 1.8 mg/cm^2 (**Supplementary Table 1**).*" (Pg. 7, Lines 6-8)

2. The specific energy and power values have been obtained from cyclic voltammetry measurements. This can be misleading. Indeed, energy and power should be evaluated by the galvanostatic discharge curves. The reason is that energy and power strongly depend on current. The Nyquist plots reported in Figure 15 show that the cells feature more than 200 ohm cm^2. This means that practical current densities for the cells assembled with MXenes-knotted CNTs cannot be higher than few mA/cm^2, that is a low value for supercapacitors.

The authors report galvanostatic tests at 10 A/g, but given that the mass loading is not given, it is not possible to get the current density. Therefore, the claim about impressive energy density and power is not very convincing.

Response: First, we would like to clarify that the mass loading has been provided in our paper.

(1) To address the concern of the reviewer on the calculation of energy density and power values, we have supplemented the energy density calculated from the GCD curves based on the equation:

$$E_{cell} = I(\int_{V_0}^{V_{max}} t\, dV) \quad \text{(R-1)}$$

In addition, the power density was further evaluated based on $P = E/t$. The power density and power values have been presented below:

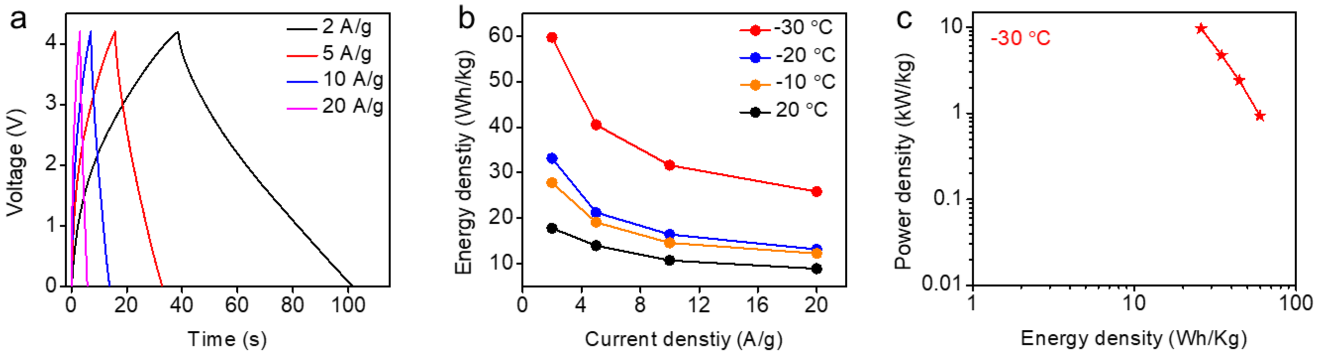

Figure R-2 (a) Galvanostatic charge/discharge curves at -30 °C, (b) energy density at different temperatures and (c) Ragone plots at -30 °C of the asymmetric cell based on the MXene-knotted CNT composite electrode with CNT content of 17%.

The current density of the galvanostatic tests was calculated from the mass of both the positive and the negative electrode (0.15 mg). As seen in Figure R-2b, the energy density is ~59 Wh/kg at 2 A/g, which is even slightly higher than value calculated from the CV curves (53 Wh/kg at 10 mV/s). In addition, a power density of 9.6 kW/kg at 20 A/g is delivered based on GCD curves, which is also higher than the value calculated from CVs (6.4 kW/kg at 500 mV/s). **It verifies our claim on the energy density and power.** Based on your suggestion, we have replaced the energy density and power density calculated from CVs in **Supplementary Fig. 17b, c** with the data in Figure R-2. The equation (3) in the manuscript has also been replaced with equation R-1. Also, the related description has been updated in the main manuscript.

(2) The high resistance is likely due to the low ionic conductivity of the electrolytes at low temperatures (Please see Figure R-3 in the next question), which is the intrinsic property of the electrolyte. As being stated in the ***Introduction*** of the manuscript (pg 2-4) as "*Efforts to improve ion accessibility for high-rate performance in organic electrolytes has been addressed using two approaches: electrolyte formulation and modification of electrode structure….., When attempting to modify electrode structures themselves to improve ion accessibility, the common approaches include introducing pores in the surface of an electrode material through chemical etching,*[10, 11] *increasing the interlayer spacing through modification of surface chemistry, or through the intercalation of pillaring materials to prevent restacking of 2D sheets.*[12-15]*…. In this study, we developed an approach to achieve high-rate performance in an organic electrolyte through the construction of a 3D electrolyte-accessible electrode structure.",* our study was focused on specifically the design and optimization of the electrode to improve the capacitance at high-rates and low temperatures. We hope that through this publication, the work can receive the strong attention from those experts working on the electrolyte formulation to solve the problem of low ionic conductivity and eventually push the field forward to the next stage together.

3. The ionic conductivity of the electrolyte at the different temperatures is not reported. This information is needed to better understand the evolution of the Nyquist plots with temperature.

Response: We have tested the ionic conductivity of the electrolyte at different temperature by using the conductivity isopod (EDAQ/EPU357). From Figure R-3, the ionic conductivity of the electrolyte decreased from 14.31 mS/cm to 0.97 mS/cm when the temperature decreases from 20 °C to -60 °C. We have added Figure R-3 in the supporting information as **Supplementary Fig. 21**.

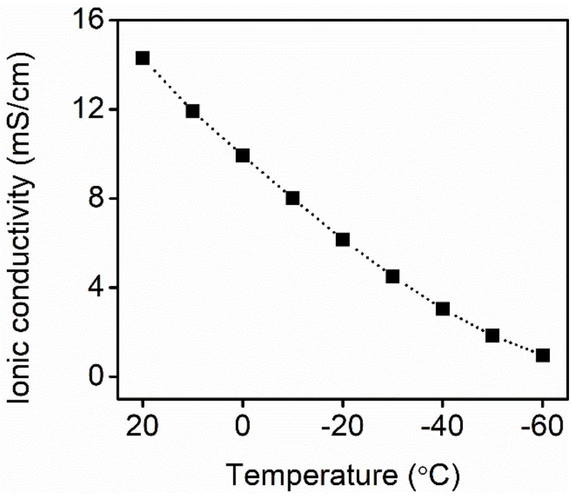

Figure R-3 Temperature dependence of the ionic conductivity for the electrolyte.

Furthermore, we have analyzed the equivalent series resistance (ESR) of our cells by electrochemical impedance spectroscopy (Figure R-4) and added further discussion to the main manuscript for the evolution of the Nyquist plots as "Nyquist plots of the cell with the 17% CNT electrode exhibited almost identical curves from 20 °C to -60 °C, indicating the temperature-independent capacitive behavior *(**Supplementary Fig. 20a**)*. Conversely, the Nyquist plots of the asymmetric cell with pure the Ti_3C_2 film as the negative electrode are severely distorted to the right at low temperatures *(**Supplementary Fig. 20b**)*. This deviation from the ideal capacitive behavior matched the rapid decrease of capacitance at lower temperatures for the asymmetric cell with pure Ti_3C_2 electrode (**Fig. 4f**). *In addition, when the temperature decreased from 20 °C to -60 °C, the equivalent series resistance (ESR) values of the cell with the 17% CNT electrode and the cell with the pure Ti_3C_2 film increased from 0.28 Ω·cm^2 to 1.91 Ω·cm^2 and from 0.24 Ω·cm^2 to 1.35 Ω·cm^2, respectively (**Supplementary Fig. 20c-d**). It can be inferred that the increase of ESR values was associated with the lowered ionic conductivity of the electrolyte at low temperatures (**Supplementary Fig. 21**).*" (Pg. 18, Lines 15-19)

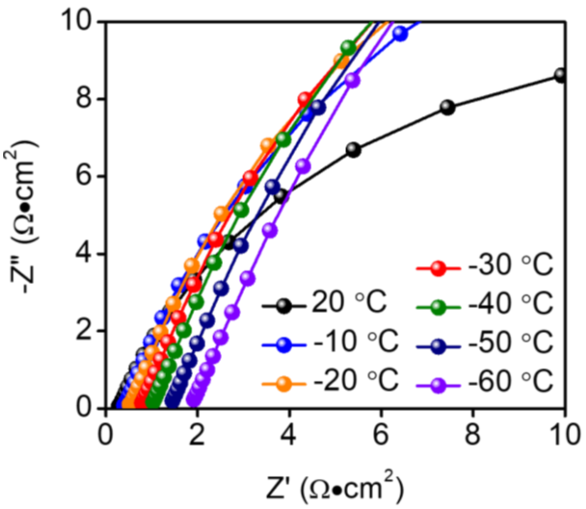

Figure R-4 Nyquist plots of the cell at different temperatures.

4. The electrochemical characterization of knotted CNTs - MXenes is presented only in the negative potential domain. I understand that this material cannot be used as positive electrode. This information is not clearly given. Furthermore, an aligned carbon nanotube electrode is used as the positive electrode, but electrochemical data (mass, specific capacitance, voltammetric reponse,…) on this electrode are not reported. These data are needed to understand the

real contribution of knotted CNTs–MXenes to the full cell performance.

Response: The electrochemical data of the aligned CNT electrode was measured and shown in Figure R-5.

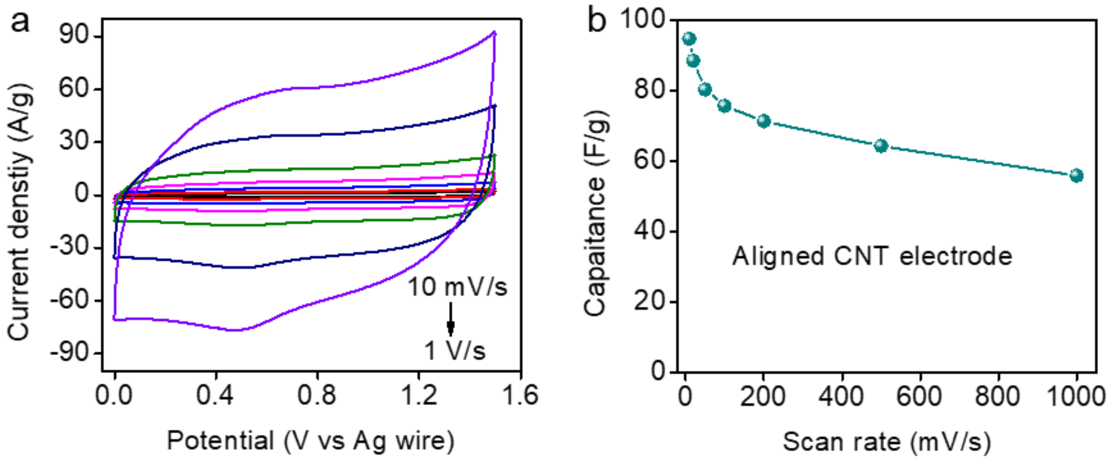

Figure R-5 (a) Cyclic voltammograms and (b) capacitance at different scan rates.

The mass loading of the aligned CNT electrode is approximately 1.4 mg/cm^2. It showed a capacitance of 94 F/g at 10 mV/s and a capacitance retention of 58% from 10 mV/s to 1 V/s. The capacitance of the asymmetric cell (C_{cell}) was obtained from the equation of $\frac{1}{C_{cell}} = \frac{1}{C_1} + \frac{1}{C_2}$ (C_1 and C_2 are the capacitance of each electrode). Since the capacitance of aligned CNT electrode is lower compared to the MXene-knotted CNT composite electrode, the MXene-knotted CNT composite electrode is dominant in the contribution of the energy storage of the cell.

We have added Figure R-5 in the supporting information as **Supplementary Figure 14** and the relevant discussion in the manuscript as below:

"*Since MXene will be oxidized at positive potentials and cannot be used as a positive electrode, we assembled asymmetric full cells with a MXene-knotted CNT composite electrode as the negative electrode and an aligned carbon nanotube electrode as the positive electrode to evaluate the performance of the MXene-knotted CNT composite electrodes at low temperatures (**Fig. 4a-c and Supplementary Figs. 14 -15**).* The aligned CNT electrode showed a working potential of 1.5 V as the positive electrode (Fig. 4a). The voltage window of the cell was maximized by balancing the mass ratio between the positive and negative electrodes. A voltage window of 3 V was realized with a mass ratio between the positive and negative electrodes of ~ 3: 2. *Since the capacitance of aligned CNT electrode (94 F/g at 10 mV/s) was lower compared to the MXene-knotted CNT composite electrode (~130 F/g at 10 mV/s), the MXene-knotted CNT composite electrode was dominant in the contribution of the energy storage of the cell (**Supplementary Fig. 14**).*" (Pg. 15, Lines 5-8 & Lines 12-14)

5. While the capacitance retention with scan rate and temperature of the materials and the full cells are interesting, the electrode and cell specific capacitance values are not outstanding. Indeed, the authors report about a cell capacitance of 20 F/g, but commercial supercapacitors typically feature 20-25 F/g. A comparison with commercial high surface area carbons tested in the same electrolyte should be reported in order to better highlight knotted CNTs-MXenes performance.

Response: First, we would like to thank the review for recognizing the achievement of our paper that "the capacitance retention with scan rate and temperature of the materials and the full cells are interesting", which is the main point of our work that we would like to share with the readers.

Second, to address the concern of the comparison of the specific capacitance values with commercial high

surface area carbons, we have prepared the electrode with the commercial activated carbon (a typical high surface area carbon) and assembled the symmetric cell for comparison. The electrode was composed of 80 wt % activated carbon (YP-50, Kuraray chemical, Japan), 10 wt % acetylene black (Alfa Aesar, 99.9%) and 10 wt % poly(tetrafluoroethylene) (PTFE) as a binder. The performance of the cell in the same electrolyte is shown as below:

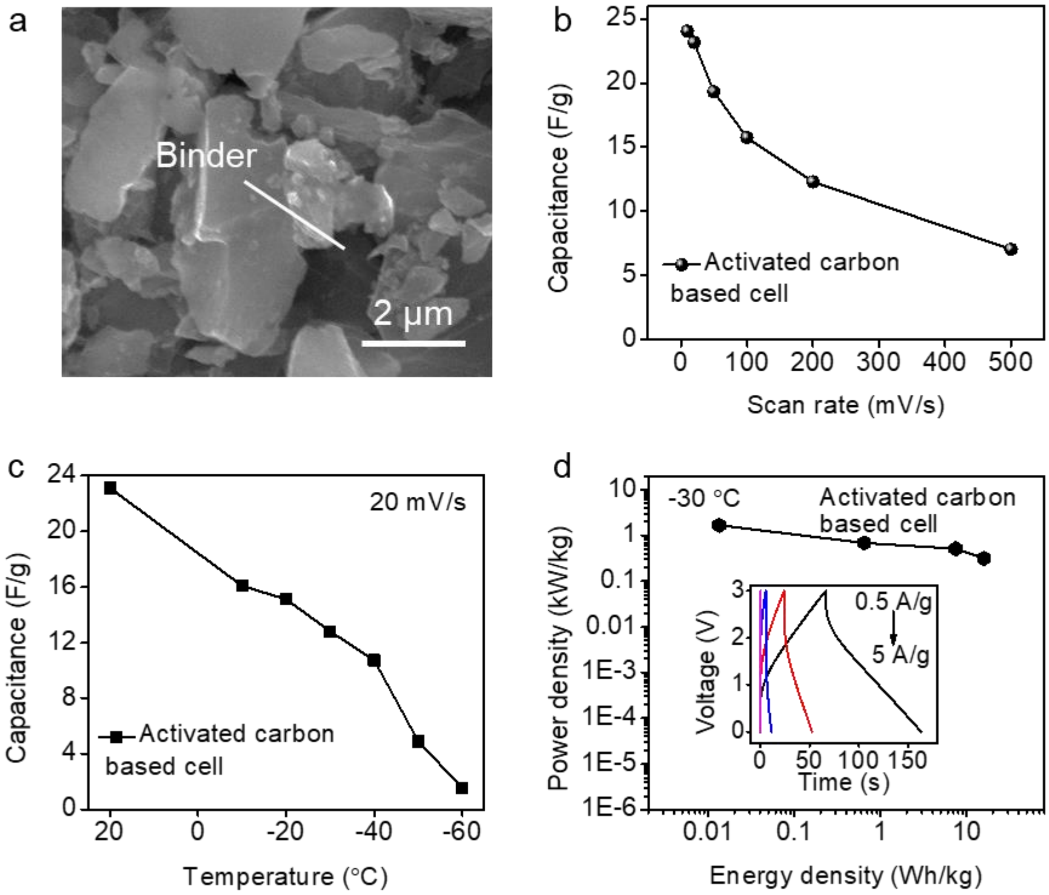

Figure R-6. (a) SEM image of the activated carbon electrode. (b) Capacitance at different scan rates, (c) capacitance at different temperatures and (d) Ragone plots at -30 °C for activated carbon based cell. Inset of Figure R-6d showed the GCD curves of the activated carbon based cell at -30 °C.

Fig. R-6a showed the structure of the activated carbon (AC) based electrode, which was composed of the AC particles with the size ranged from 0.5~5 μm. Due to the geometric discontinuity of AC particles, the binder (PTFE) and acetylene black had to be used to improve the conductivity of the electrode. The AC-based symmetric cell showed a capacitance of 24 F/g at 10 mV/s and a capacitance retention of 29% from 10 mV/s to 500 mV/s (Figure R-6b). In addition, the capacitance of the AC-based symmetric cell decreased from 23 F/g to 1.5 F/g at 20 mV/s when the temperature decreased from 20 °C to -60 °C (Figure R-6c). As seen in **Supplementary Figure 17**, our cell has the capacitance of 23 F/g, which is comparable with AC-based cell. Besides, our cell has much improved capacitance retention at increased scan rates (50% from 10 mV/s to 500 mV/s) and at low temperatures (55% from 20 °C to -60 °C) (**Figure 4f**). Moreover, our cell had a larger voltage window (4.2 V) than that of the AC-based symmetric cells (3 V) at low temperatures, which delivered energy density of 59 Wh/kg that was almost 4 times of the value of the AC-based cell (~15 Wh/kg) (Figure R-6d). It is worth noting that the usage of binders always involves the impurity into electrodes, causing the limitation to realize high voltage and high energy density, which emphasizes the advantages of the production of binder-free freestanding electrode as exemplified by our knotted-CNT/Mxene electrode.

Based on the comparison, we can conclude that **while our 3D electrolyte-accessible architecture electrode has the comparable room-temperature performance with the commercial materials (e.g. active**

carbon), the high-rate capacitance and low-temperature performance are both record high in the related field. It indicates that the strategy of our work to realize the high rate and low temperature operation of supercapacitors through the modification of electrode structure is successful and effective. We hope the results would broadly lead to a better understanding of how supercapacitor devices work at high scan rates and low temperatures. In addition to the development of low-temperature electrolyte, structural design of electrodes would be another route to enable the low-temperature operation of supercapacitors.

We thank for the reviewer's suggestions and have added Figure R-6 as **Supplementary Figure 18** and more discussion in the manuscripts to highlight the advantages of our electrodes compared with commercial carbon electrodes. The discussions are listed as: "*We compared the performance of our MXene-knotted CNT composite electrode electrodes based on commercial high surface area activated carbon (AC). The AC-based symmetric cell showed a capacitance of 24 F/g at 10 mV/s and a capacitance retention of 29% from 10 mV/s to 500 mV/s. In addition, the capacitance of the AC-based symmetric cell decreased from 23 F/g to 1.5 F/g at 20 mV/s when the temperature decreased from 20 °C to -60 °C (**Supplementary Fig. 18**). As seen in **Supplementary Figure 17**, our cell has the comparable capacitance of 23 F/g with AC-based cell, yet has much improved capacitance retention at increased scan rates (50% from 10 mV/s to 500 mV/s) and at low temperatures (55% from 20 °C to -60 °C) (**Figure 4f**). Moreover, our cell had a larger voltage window (4.2 V) than that of the AC-based symmetric cells (3 V) at low temperatures, which delivered energy density of 59 Wh/kg that was almost 4 times of the value of the AC-based cell (~15 Wh/kg).*" (Pg.17, Lines 14-20 & Pg.18, Lines 1-3)

6. Additional data (like XPS) on the surface chemistry of the knotted CNTs - MXenes should be reported to exclude any pseudocapacitive contribution that could be related to surface moieties.

Response: We have tested the XPS of the MXene-knotted CNT electrodes and the results are shown below:

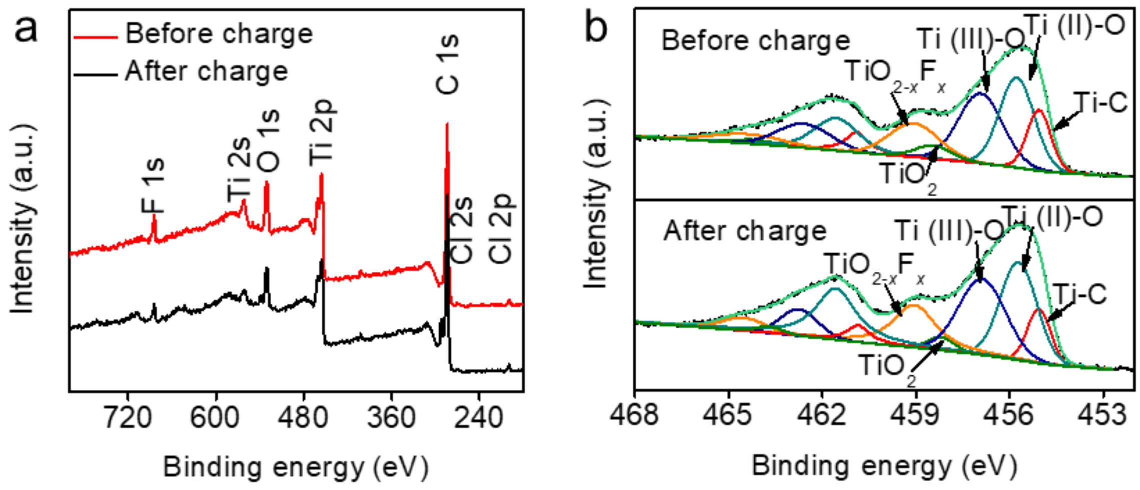

Figure R-7 XPS spectra of the MXene-knotted CNT composite electrode before and after charge. (a) General survey scan of the surface and (b) deconvolution of the Ti 2p peaks.

From the general survey scan of the surface, the MXene composite electrodes have surface function groups of =O, -OH, -Cl and -F. The Ti $2p_{3/2}$ peak at 455.0 eV is associated with Ti bonded to C. The Ti $2p_{3/2}$ peaks at 455.8 eV and 457.1 eV correspond to the Ti atoms bonded to O. The Ti $2p_{3/2}$ peak at 458.6 eV is associated with TiO_2, while the Ti $2p_{3/2}$ peak at 459.2 eV corresponds to $TiO_{2-x}F_x$. No significant change of Ti 2p peaks could be observed before and after charge, which excluded any pseudocapacitive contribution during the electrochemical process. The charge storage mechanism in non-aqueous electrolytes should follows the intercalation mechanism with little pseudocapacitive contribution (*J. Power Sources* **2016**, *306*, 510-515; *J. Power Sources* **2016**, *326*, 575-579; *Nat. Energy* **2019**, *4*, 241-248), which has also been proved by molecular

dynamics simulation (*ChemSusChem*, **2018**, *11*, 1892-1899).

We have added Figure R-7 in the supporting information as **Supplementary Fig. 11** and add descriptions about the surface chemistry in the manuscript: "*Additionally, the XPS spectra of the MXene-knotted CNT electrodes before and after cycling showed that the surface chemistry was not noticeably altered, indicating that the electrochemical reactions did not significantly affect the surface chemistry of the MXene in this system (Supplementary Fig. 11).*" (Pg. 14, Lines 7-10)

Minor comments:

Page 2. "organic electrolytes have larger charge storing ions ". I suggest to change it with "organic electrolytes have larger ions" and to add a comment about different solvation shells of ions in organic solvents and water.

Response:

Agreed. We have changed this sentence and added the comments in the introduction.

"*However, organic electrolytes have larger ions and lower conductivity than aqueous electrolytes. Besides, the solvation shells of ions in organic electrolytes also have a larger size than those in aqueous electrolyte. These properties hinder charge storage and slow the ion transport kinetics of electrode materials, limiting high-rate performances, especially at low temperatures.*" (Pg. 2 Lines 10-14)

-Figure 4b. It is not correct to report the y-axis in terms of specific capacitance because specific capacitance has not negative values. Voltammetries should be reported in terms of current density or specific current.

Response: The negative values of capacitance in the CVs are not "negative", per se. The transformation of current to specific capacitance is a simple arithmetic transformation: gravimertric capacitance (F/g, A×s/V×g) = current (i)/(scan rate (mV/s)×electrode mass(mg)). So, the "negative" capacitance (current) is simply the discharge capacitance (current). This method is especially helpful for pseudocapacitve materials where redox peaks are usually only apparent at low scan rates and would not be visible in the current density based voltammograms. Indeed, the method has become extremely common to present voltammograms with specific capacitance as the y-axis, since this allows for fair comparison between differing materials and electrodes with differing mass loading (*Nat. Energy* **2019**, *4*, 241-248; *Nature* **2018**, *557*, 409-412).

To address your concern, we have plotted the data with the y-axis in terms of current density in the supporting information, where the plots of the CVs at different scan rates and different temperatures are shown in **Supplementary Fig. 15**. These plots are helpful to analyze the electrochemical behavior at high rates.

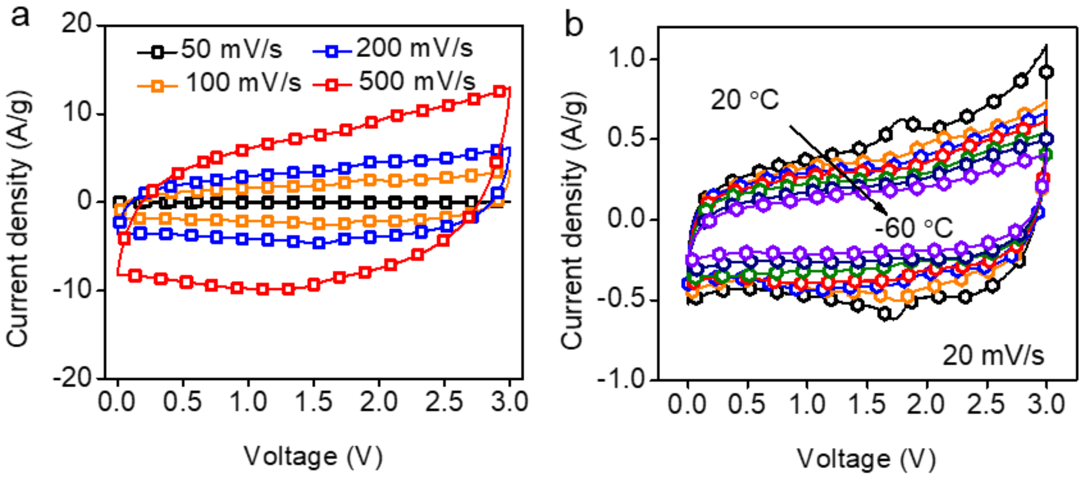

Figure R-8 (a) CVs at different scan rates and (b) CVs at different temperatures for the asymmetric cells based on the MXene-knotted CNT composite electrode (17% CNT content)

-Page 19. Energy and power should be calculated from the galvanostatic tests. In any case, these values should refer to the discharge. In case of the voltammetric analyses of the full cell, they should be obtained from the cathodic scan (negative currents) and not from the anodic scan.

Response: We have supplemented the energy density and power density calculated by using the GCD curves (Figure R-2) (Please check the response to your Question 1). The energy and power densities obtained from the charge calculated using the voltammogram is reasonable.

Regarding your concern of the voltammetric analyses of the full cell, we apologize that we didn't clarify the difference between the calculation of the capacitance between the 3-electrode cells and the 2-eletrode cells in the Methods section in the previous version. Now, we have clarified this portion as "*For the two-electrode cell, the capacitance of the full cell (C_{cell}) was evaluated based on the CV curve from the cathodic scan of CV curve*" (Pg. 23, Lines 15-16).

Reviewer #3 (Remarks to the Author):

The authors have studied and reported the use of a knotted CNT for disrupting the alignment of the Ti_3C_2 flakes, leading to increased ion transport pathways and ion accessibility. The work is quite interesting as it also deals with storage devices working at sub-zero temperatures. However, the following clarifications with some minor corrections are necessary for the manuscript to be considered for publication.

Response: Thank you for your time and valuable comments on our work, please see our detailed response below.

1. The authors are advised to perform the spell check thoroughly. Ex: 'remaines' in introduction. In addition, there is a mixture of past and present tenses at some parts.

Response: We have corrected the error. We have asked a native English speaker to proofread our manuscript and to improve the readability.

2. Does the growth of thick and thin CNTs depends on the temperature shift or with the use of different sized catalysts? Do both have an effect? Kindly clarify.

Response: Both the temperature shift and the formation of different sized catalysts influence the synthesis of the knotted CNTs.

Please allow us to clarify the synthesis process. First, the Ni-Mn-Al-O nanoparticles were synthesized using a typical co-precipitation and subsequent calcination reaction (*Details in Catalyst Preparation of Experimental section*). Then the nanoparticles were loaded into the reactor and processed with our customed two-step growth (**Supplementary Fig. 2**). The first-step growth was held in the upper zone of the reactor with the temperature of 750 °C. The nanoparticles were immersed in the H_2 (flow rate of 80 mL/min) for 10 minutes for the catalyst formation and then C_2H_2 (flow rate of 100 mL/min) was introduced for growing CNTs for 15 minutes. During the first-step growth, the large-sized catalysts were conceived from the nanoparticles and thick CNTs were acquired with the average diameter of ~55 nm (**Supplementary Fig. 3a**). Then the isolation board was opened and the nanoparticles (with the thick CNTs on them) were moved to the lower zone of the reactor with the temperature of 950 °C. At this stage, the small catalysts were conceived from the nanoparticles and the thin CNTs were grown with the average diameter of ~ 15 nm. The second-step growth would be held for another 15 minutes and the knotted structured CNTs with a bimodal diameter distribution were obtained (**Supplementary Fig. 3b**).

Quick summary: **1.** The usage of temperature-shifting two-stage fluidized bed reactor with separate temperature zones is the key for the growth of the knotted CNTs, which can provide the specific synthesis

condition for the formation of large/small sized catalysts and the growth of thick/thin CNTs separately. **2.** Considering the knotted structure with the thin CNTs intertwining on the thick CNTs, the thick CNTs should firstly grow on the catalyst following Figure 1a. Thus, the temperature-shifting two-stage fluidized bed reactor is helpful to control the growth sequence to finally realize the growth of knotted CNTs.

3. The authors have mentioned that by varying the size of the catalyst, different sized CNTs were obtained. How have the authors varied the size of the catalyst and how is it confirmed? The methods section do not discuss on obtaining various sized catalysts.

Response: As addressed to in the previous question, the variation of the size of the catalysts was realized through the temperature shift in our customed two-stage fluidized bed reactor with separate temperature zones. While the lower temperature (750 °C) was applied to conceive the large-sized catalysts for obtaining the thick CNTs with the average diameter of ~55 nm, the higher temperature (950 °C) was applied to conceive the small-sized catalysts for obtaining the thin CNTs with the average diameter of ~15 nm.

As shown in **Supplementary Fig. 3a,** we investigated the tube diameter distribution of the CNTs after first-step growth in the upper zone (750 °C) through SEM observation and confirmed that the thick CNTs were obtained with the diameter of 55 ± 5nm. When the two-step growth was completed, we investigated the tube diameter distribution and confirmed that the thin CNTs with the diameter of 15 ± 8 nm were obtained during the second-step growth (**Supplementary Fig. 3b)**.

We have supplemented the description of **Supplementary Fig. 2&3** in the Methods section as "*0.2 g Ni-Mn-Al-O nanoparticles were loaded into the reactor and processed with our customed two-step growth (**Supplementary Fig. 2**). The first-step growth was held in the upper zone of the reactor with the temperature of 750 °C. The nanoparticles were immersed in the H_2 (flow rate of 80 mL/min) for 10 minutes for the catalyst formation and then C_2H_2 (flow rate of 100 mL/min) was introduced for growing CNTs for 15 minutes. During the first-step growth, the large-sized catalysts were conceived from the nanoparticles and thick CNTs were acquired with the average diameter of ~55 nm (**Supplementary Fig. 3a**). Then the isolation board was opened and the nanoparticles (with the thick CNTs on them) were moved to the lower zone of the reactor with the temperature of 950 °C to proceed the second-step growth for another 15 minutes. During this process, the small catalysts were conceived from the nanoparticles and the thin CNTs were grown with the average diameter of ~ 15 nm (**Supplementary Fig. 3b**). Then the knotted structured CNTs with a bimodal diameter distribution were obtained.*" (Pg. 20, Lines 17-21 & Pg. 21, Lines 1-6)

4. The authors could discuss on the mechanical stability of the knotted CNTs at large cycles.

Response: Thank you very much for your suggestion. We have added the discussion on the mechanical stability of the knotted CNTs at large cycles as "*As addressed above, the structural stability of the knotted CNTs can even withstand sonication due to the mechanical constraints (**Fig. 2b**). The knotted CNTs were capable of maintaining the structural integrity of the electrode film by preventing the restacking of the MXene flakes upon charge/discharge, which guaranteed the efficient ion transportation during long term cycling.*" (Pg. 14, Lines 18-21)

5. The size of the CNT knots is mentioned to be 200 ± 20 nm and that of Ti_3C_2 flakes as ~250 nm. How have the authors confirmed the exact size of these particles?

Response: We confirmed the size the flakes and knots from SEM characterization. We have measured the sizes from numerous SEM images and statistically calculate the data. The size can be easily confirmed from the SEM images of the knotted CNTs in Figure 2 and the Ti_3C_2 flakes in **Supplementary Figure 6**.

6. Details on the aligned CNT used as positive electrode in ASC could be discussed.

Response: The electrochemical data of the aligned CNT electrode was measured and shown in Figure R-5.

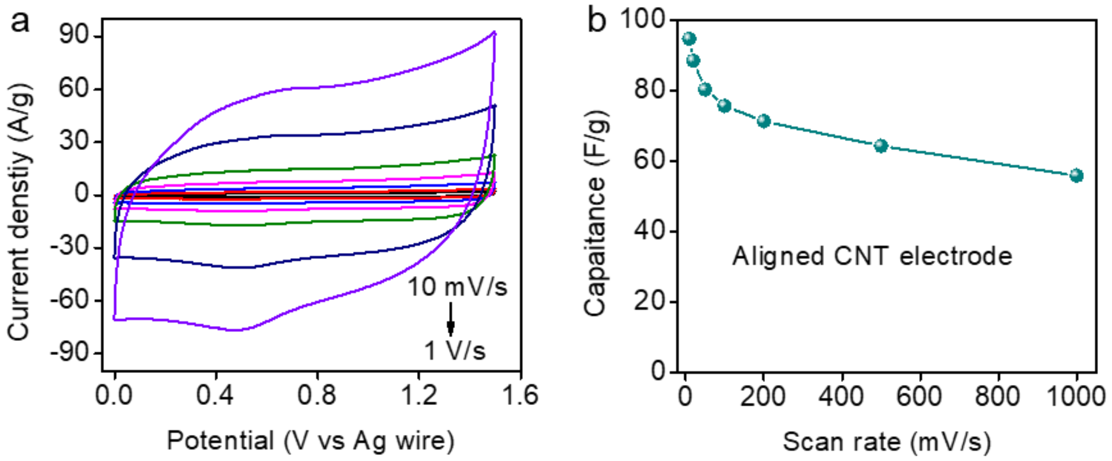

Figure R-5 (a) Cyclic voltammograms and (b) capacitance at different scan rates.

The mass loading of the aligned CNT electrode is approximately 1.4 mg/cm^2. It showed a capacitance of 94 F/g at 10 mV/s and a capacitance retention of 58% from 10 mV/s to 1 V/s. The capacitance of the asymmetric cell (C_{cell}) was obtained from the equation of $\frac{1}{C_{cell}} = \frac{1}{C_1} + \frac{1}{C_2}$ (C_1 and C_2 are the capacitance of each electrode). Since the capacitance of aligned CNT electrode is lower compared to the MXene-knotted CNT composite electrode, the MXene-knotted CNT composite electrode is dominant in the contribution of the energy storage of the cell.

We have added Figure R-5 in the supporting information as **Supplementary Figure 14** and the relevant discussion in the manuscript as below:

"*Since MXene will be oxidized at positive potentials and cannot be used as a positive electrode, we assembled asymmetric full cells with a MXene-knotted CNT composite electrode as the negative electrode and an aligned carbon nanotube electrode as the positive electrode to evaluate the performance of the MXene-knotted CNT composite electrodes at low temperatures (**Fig. 4a-c and Supplementary Figs. 14-15**).* The aligned CNT electrode showed a working potential of 1.5 V as the positive electrode (Fig. 4a). The voltage window of the cell was maximized by balancing the mass ratio between the positive and negative electrodes. A voltage window of 3 V was realized with a mass ratio between the positive and negative electrodes of ~ 3: 2. *Since the capacitance of the aligned CNT electrode (94 F/g at 10 mV/s) was lower compared to the MXene-knotted CNT composite electrode (~130 F/g at 10 mV/s), the MXene-knotted CNT composite electrode was dominant in the contribution of the energy storage of the cell (**Supplementary Fig. 14**).*" (Pg. 15, Line 5-8 & Line 12-14)

7. The authors could compare the electrochemical performance of MXene with the MXene-CNT composite.

Response: We have compared the electrochemical performance of MXene with the MXene-CNT composite in our paper, but the labeling in our figures may not have communicated this clearly. The "pure film" in **Fig. 2g** in the original manuscript is the MXene electrode without any knotted CNTs. We have replaced the label "pure film" in **Fig. 2g** and caption with "*pure Ti_3C_2 film*" to avoid further misunderstandings. Meanwhile, as seen in **Fig. 3a**, the MXene-CNT composite electrodes showed much higher capacitance and better rate performance compared to the pure Ti_3C_2 electrodes.

Reviewer #2 (Remarks to the Author):

The authors have improved the manuscript even by including additional data and clearly addressed my previous comments. Therefore, I suggest publication of this paper. However, still few revisions are needed before publication:

1. Equation 3 is not correct: Energy is given by the integral of the cell voltage over capacity, i.e. at constant current E=I x Int (V dt) and not E= Ix int (t dV)
2. The sentence “Since the capacitance of the aligned CNT electrode (94 F/g at 10 mV/s) was lower compared to the MXene-knotted CNT composite electrode (~130 F/g at 10 mV/s), the MXene-knotted CNT composite electrode was dominant in the contribution of the energy storage of the cell” is not fully correct. Indeed, the energy response of a cell is limited (dominated) by the lowest performance electrode.
1. I know that it has become extremely common to present voltammograms with specific capacitance as the y-axis. And this is a problem! Indeed, this does not mean that this is also correct. Therefore, I strongly invite the authors to substitute in the main text all the voltammograms and to report the data in terms of current density or specific current.

Reviewer #2 (Remarks to the Author):

The authors have improved the manuscript even by including additional data and clearly addressed my previous comments. Therefore, I suggest publication of this paper. However, still few revisions are needed before publication:

Response: We thank the reviewer's support and valuable comments on our work, please see our detailed response below.

1. Equation 3 is not correct: Energy is given by the integral of the cell voltage over capacity, i.e. at constant current E=I x Int (V dt) and not E= Ix int (t dV).

Response: We have corrected the equation as below:

$$E_{\text{cell}} = I\left(\int_0^{t_\text{d}} V\,\text{d}t\right) \qquad \text{(R-1)}$$

where I was the discharging current density, t_d was the discharging time, V was voltage of the asymmetric cell. The revision has been made in the Page 24, lines 12-14 in the manuscript.

2. The sentence "Since the capacitance of the aligned CNT electrode (94 F/g at 10 mV/s) was lower compared to the MXene-knotted CNT composite electrode (~130 F/g at 10 mV/s), the MXene-knotted CNT composite electrode was dominant in the contribution of the energy storage of the cell" is not fully correct. Indeed, the energy response of a cell is limited (dominated) by the lowest performance electrode.

Response: We have revised the sentence as "*The capacitance of the aligned CNT electrode (94 F g^{-1} at 10 mV s^{-1}) was found to be lower than that of the MXene-knotted CNT composite electrode (~130 F g^{-1} at 10 mV s^{-1}) (Supplementary Fig. 14). Since the capacitance of the cell was limited by the lower-capacitance electrode, the aggregate capacitance can be further improved by using the positive electrode possessing higher capacitance.*" (Page 15 lines 20-21 & Page 16 lines 1-2)

3. I know that it has become extremely common to present voltammograms with specific capacitance as the y-axis. And this is a problem! Indeed, this does not mean that this is also correct. Therefore, I strongly invite the authors to substitute in the main text all the voltammograms and to report the data in terms of current density or specific current.

Response: We have replaced the voltammograms in the main text (including Fig. 3d, inset of Fig. 3f, Fig. 4a-b and Fig. 4d-e) with specific current as y-axis. In addition, we have moved the original Fig. 3d and Fig. 4b, e (the plots with capacitance as y-axis) to the supporting information as Supplementary Fig. 8d and Supplementary Fig. 15, respectively. The description and figure caption have been revised accordingly and highlighted in the text.
